# Supplementary material for: Intelligent wearable olfactory interface for latency-free mixed reality and fast olfactory enhancement
Source: Nat Commun. 2024 May 25;15:4474. doi: 10.1038/s41467-024-48884-z (PMC11128017; doi:10.1038/s41467-024-48884-z)
Supplement: Supplementary file 1 — Supplementary Information [file 41467_2024_48884_MOESM1_ESM.pdf]

Supplementary Information

Intelligent Wearable Olfactory Interface for Latency-Free Mixed Reality and Fast Olfactory Enhancement

Yiming Liu[1,2,10], Shengxin Jia[1,3,10], Chun Ki Yiu[1,3,10], Wooyoung Park[1,10], Zhenlin Chen[1,3], Jin Nan[4], Xingcan Huang[1], Hongting Chen[2], Wenyang Li[1], Yuyu Gao[1], Weike Song[5], Tomoyuki Yokota[2,6], Takao Someya*[2,7,8], Zhao Zhao*[5], Yuhang Li*[4], and Xinge Yu*[1,3,9]

[1]Department of Biomedical Engineering
City University of Hong Kong
Kowloong Tong (Hong Kong)
[2]Department of Electrical Engineering and Information systems
The University of Tokyo
Tokyo 113-8656 (Japan)
[3]Hong Kong Center for Cerebra-Cardiovascular Health Engineering
Hong Kong Science Park
New Territories 999077 (Hong Kong)
[4]School of Aeronautic Science and Engineering
Beihang University
Beijing 100191 (China)
[5]China Special Equipment Inspection and Research Institute
Beijing 100029 (China)
[6]Institution of Engineering Innovation
The University of Tokyo
Tokyo 113-8656 (Japan)
[7]RIKEN Center for Emergent Matter Science (CEMS)
Saitama 351-0198 (Japan)
[8]Thin-film Device Laboratory
RIKEN
Saitama 351-0198 (Japan)
[9]Hong Kong Institute for Clean Energy (HKICE)
City University of Hong Kong, Hong Kong, China
[10]These authors contributed equally: Yiming Liu, Shengxin Jia, Chun Ki Yiu, and Wooyoung Park

E-mail:_ someya@ee.t.u-tokyo.ac.jp (TS); zhaozhao@csei.org.cn (ZZ); liyuhang@buaa.edu.cn (YL); xingeyu@cityu.edu.hk (XY)

Keywords: Olfaction interface, mixed reality, human machine interfaces, wearable electronics, olfactory training

Table S1. Comparison of our work with the olfactory interfaces reported recently.

**Working principle**	**Response time (s)**	**Size, L × W × H (mm^3)**	**Number of supply odors**	**Power consumption (mW)**	**Applications**	**Reference**
Piezoelectric atomizer	N/A	Diameter of 14 mm, and length of 56.8 mm for the container	1	N/A	Influence emotions and cognitive performance	3
Vaporization odorous chemicals by heating, then delivered by fan	A few seconds	84 × 59 × 31	8	N/A	Influencing emotions, and alerting upcoming event	14
Physical phase change of odorous paraffin wax	30	130 × 110 × 80	6	N/A	Olfaction training	15
Atomizer	6	110 × 56 × 35	1	N/A	VR game	16
Blowing odor vapor by fans	> 1	527 × 60 × 328	1	N/A	VR game	24
Atomizer	5.16	N/A	4	N/A	VR game	25
SAW atomizer	6	Length, over 20 cm	8	N/A	Multimedia content and VR	26
Physical phase change of odorous paraffin wax	1.4 s	18 × 16 × 3 for each OG	9	220.4	VR, 4D movie, smell message delivery, clinical treatment, and emotion smoothing.	Our previous work, 2
Physical phase change of odorous paraffin wax	1 s	11 × 10 × 2.2 for each OG	8	317.4	Message delivery	Our previous work, 4
Solenoid valve	0.07 s	11 × 10 × 1.8 for each OG	32	84.8	MR and olfaction training.	This work

Supplementary Notes

1. Circuit operation of 2-channel OGs based olfaction interface. The two-channel control panel is operated by an external 10 V battery while by applying two low-dropout regulators (LDOs) and a voltage divider, 5 V (LP38692SDX-5.0/NOPB, Texas Instruments Inc.), 3.3 V (LP3990MFX-3.3/NOPB, Texas Instruments Inc.), and 2 V are supplied to a microcontroller unit (MCU) (ATMEGA328P-MU, Microchip Technology Inc.) for controlling two OGs and processing data, to a Bluetooth module (WH-BLE106, Jinan USR IOT Technology Limited) for wireless communication among the device, VR equipment, and computer, and to decoders (SN74LVC1G19DBVR, Texas Instruments Inc.) for controlling the opening and closing of the breathing holes of the OGs, respectively (**Supplementary Fig. 13**). Following, when the target temperature and duty cycles of the opening and closing breathing holes are established, by switching on and off a NMOS (SI2336DS-T1-GE3, Vishay Intertechnology, Inc.), electrical power is applied to the heating electrode for stabilizing the heating temperature at the specific target one. At the same time, the heating temperature is monitored by the thermistors embedded in the heating electrodes. By applying a 24-bit analog-to-digital converter (ADC) (ADS1220IRVAR, Texas Instruments Inc.), a higher resolution temperature sensing system can be achieved. On the other hand, by inputting high and low digital signals alternately to the decoders based on the settled duty cycle, the directions of the current flows into each Cu coil are shifted. Thus, the directions of the forces induced by the electromagnetic field are switched accordingly for manipulating the status of breathing holes.

2. Circuit operation of 32-channel OGs based olfaction interface. As shown in **Supplementary Fig. 20**, for operating the 32-channel OGs based olfaction interface, a MCU (ATMEGA328P-MU, Microchip Technology Inc.) is adopted to control two sets of an array of four shift registers (SN74HC595BRWNR, Texas Instruments Inc.) for controlling the heating electrode and the direction of the electromagnetic field of the magnets installed in the OGs respectively. Meanwhile, the heating temperatures are measured through four multiplexers (MAX4691EGE+T, Analog Devices Inc.) and a 24-bit ADC module (ADS1220IRVAR, Texas Instruments Inc.) connected to the MCU via SPI for higher analog reading resolution. The whole system is managed by an external battery (12 V) while four different power adjusting modules which are three voltage adjustable LDOs for operating the MCU (AZ1084CD-5.0TRG1, Diodes Inc., 5 V), heating electrodes (AZ2185D-ADJTRG1, Diodes Inc., from 0 to 12 V), and a Bluetooth module (WH-BLE106, Jinan USR IOT Technology Limited) (LP3990MFX-3.3/NOPB, Texas Instruments Inc., 3.3 V) and a DC/DC converter for generating power for the electromagnetic field (TPSM53602RDAR, Texas Instruments Inc., 2 V). Accordingly, when the device is turned on, the system is booted with a stand-by setting that all the heating electrodes of 32-channel OGs are stabilized at 35 °C and the breathing holes at the top PET layers are closed by the PET cantilever controlled by the electromagnetic field induced by a magnet and a coil. To establish this condition, firstly, the temperatures of each heating electrodes are monitored by the thermistors placed in the middle of heating electrodes while four multiplexers providing 32 analog reading channels in total are connected to each four analog reading pins of the 24-bit ADC module for increasing channels and high-resolution temperature sensing. Each multiplexer output line is connected with a reference resistor to construct a voltage dividing circuit for exploiting the thermistors as temperature sensors. Then, according to the thermal information sensed

by the sensors, the heating electrodes are turned on and off to stabilize their temperature at the fixed target by switching NMOS (SI2336DS-T1-GE3, Vishay Intertechnology, Inc.) that is controlled by an array of four shift registers (eight bits each). When the corresponding bit of a heating electrode is high, the NMOS is switched on for enabling a current flow from the 9 V power source to the heater and vice versa. Similar to the manipulation of the heaters, to control the PET cantilevers for closing and opening the breathing holes, the four-shift register array is managed by the MCU. The output signals from each shift register pin are directly connected to the corresponding decoders (SN74LVC1G19DBVR, Texas Instruments Inc.) for altering the direction of the current flow through each coil (2 and -2 V respectively). Therefore, according to the current flow directions, the directions of the forces induced by the electromagnetic field are manipulated for closing and opening the breathing holes of each OG unit. In addition, the whole system including the selections of OGs for operation, controlling the opening and closing duty cycles of the breathing holes (0, 20, 40, 60, 80, and 100%), and the target heating temperatures (35, 45, and 55 °C) are controlled wirelessly through the Bluetooth module in real-time via the self-developed GUI run by an external device such as a computer. At the same time, the temperature of the selected heater is illustrated on the GUI according to the data transmitted wirelessly from the device (**Movies S4 and S5**).

3. LRF recommendation system action generation

The list-wise recommending procedure consists of two steps, 1) scoring steps, and 2) action-generating steps. Since the olfactory test results include two parts: recognition rate $y_t^k \sqsubseteq \{-1,\ 1\}$, and the decision time Δt_t for a single order. We define the scoring function $g(y_t^k, \Delta t_t^k)$ as a weighted sum of evaluation of both y_t^k and Δt_t^k, as shown here:

$$g(y_t^k, \Delta t_t^k) = w_1 y_t^k + w_2 \left(max(0,\ y_t^k) - \min\left(1, max\left(0, (\Delta t_t^k - \Delta t_{min}) \ / \ (\Delta t_{max} - \Delta t_{min})\right)\right)\right)$$

Equation 4

Where Δt_{max} and Δt_{min} are the maximum and minimum reaction time from the initial test s_0. We set weights w_1 and w_2 as 0.75 and 0.25 which pushes scores to the range of (0.75, 1) if the users recognize the odor, and (-0.75, -1) if they fail. In other words, we can infer the recognition rate if the score is higher than 0.75 or lower than -0.75. After recommender agent scores all odors based on current states, the agent assigns training time to each odor in such a way that maximizes expected score improvement within limited time. The expected recognition rates are calculated based on the action-value function or Q values while we assume that the expected reaction time improves 10% after every 0.5 min of training. For simplicity, we use discrete training time with incremental changes of 0.5 min. The best combination of action is optimized as a modified assignment problem using linear programming in PuLP.

Critic agent. We apply the Sarsa method to find the optimal action-value function $Q(y_t, a_t)$, the expected return based on state s_t and the action a_t. Then, according to $Q(s_t, a_t)$, the agent generates proper actions in the following interactions to improve performance. As a Temporal Difference method, the optimal Q values can be found by iteratively updating the estimate $Q(y_t, a_t)$. The updated rule of Sarsa is shown here:

$$Q(y_t, a_t) \leftarrow Q(y_t, a_t) + \alpha[r + \gamma Q(y_{t+1}, a_{t+1}) - Q(y_t, a_t)]$$

Equation 5

We initialize Q values with the assumption that the recognition rate improves 10% after every 0.5 min of training. Thus, for an unrecognized odor, the expected return will jump from -0.75 to 0.75 after training about 4 min like a clamp function.

4. Illustration of the five tuples (*S, A, R, P, γ*)

- State space, *S*: $s_t = \{s_t^1, \ldots s_t^k\} \in S,\ s_t^k = [y_t,\ \Delta t_t]^k$ is defined as the test results: recognition rate $y_t^k \sqsubseteq \{-1, 1\}$, and the decision time Δt_t^k for all *k* odors at time *t*.
- Action space, *A*: $a_t = \{a_t^1, \ldots a_t^k\} \in A,\ a_t^k \geq 0,$ is to recommend a list of training times for *k* odors at time *t*.
- Reward *R*: After the user finished the olfactory training according to the recommendation from the recommender agent, an odor recognition test would be conducted by utilizing the flexible, high-channel olfaction interface system, then scores would be calculated based on the test results. The reward is defined to relate to the recognition that differs from the previous test state, which not only encourages flipping on unrecognized odors, but also prioritizes the total number of recognizable odors in the final test.
- Transition probability, *P*: Transition probability $p(s_{t+1}/s_t, a_t)$ defines the probability of state transition from s_t to s_{t+1} when the user takes actions following the recommendation a_t. We assume that the Markov Decision Process satisfies $p(s_{t+1}/s_t, a_t, \ldots s_1, a_1) = p(s_{t+1}/s_t, a_t)$. Although it may not be true all the time, we assume if the user takes zero training for *k* odor, then the next state $s_{t+1}^k = s_t^k$.
- Discount factor, *γ*: $\gamma \in [0,\ 1]$ defines the discount factor for future rewards. In particular, with $\gamma = 0$, recommender agent only focuses on the immediate reward. On the contrary, when $\gamma = 1$, the agent focuses more on future rewards.

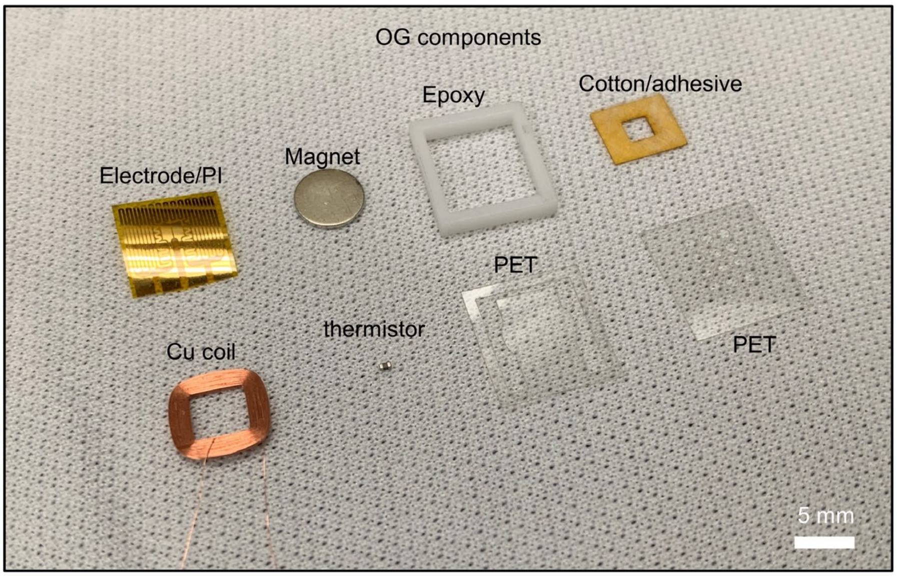

Fig. S1. Optical image of the OG components, including a photoetched heating electrode based on a 25-μm PI film, a magnet coil, a supporting Epoxy ring, a cotton layer, a copper coil, a thermistor, a PET cantilever, and a PET film with reserved holes.

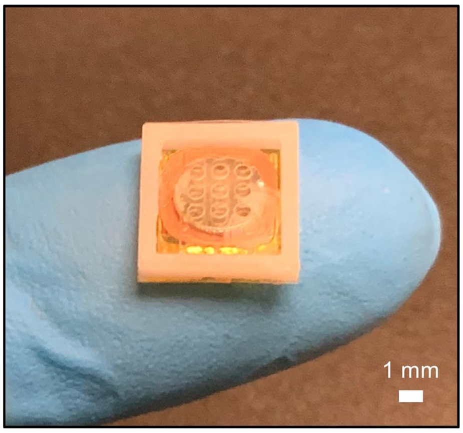

Fig. S2. Optical image of the OG mounted on the human finger.

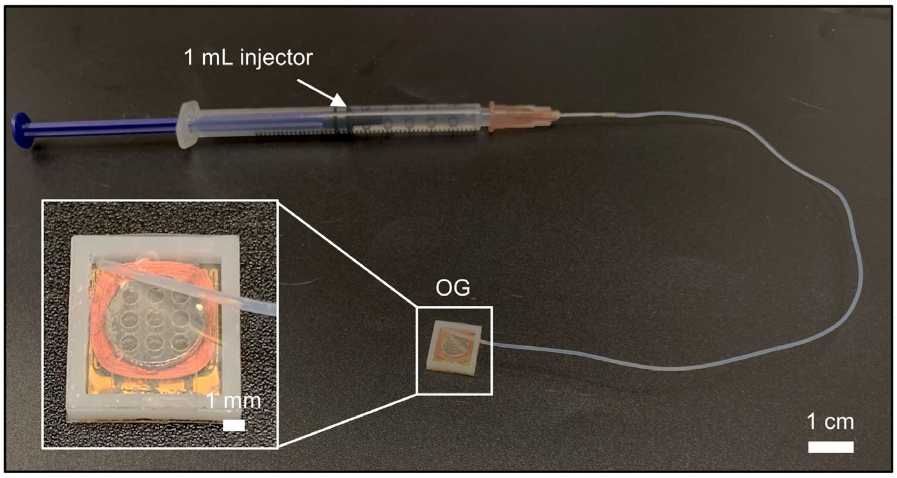

Fig. S3. A method for adding liquid perfume into the OG through the breathing holes with the enlarged detail.

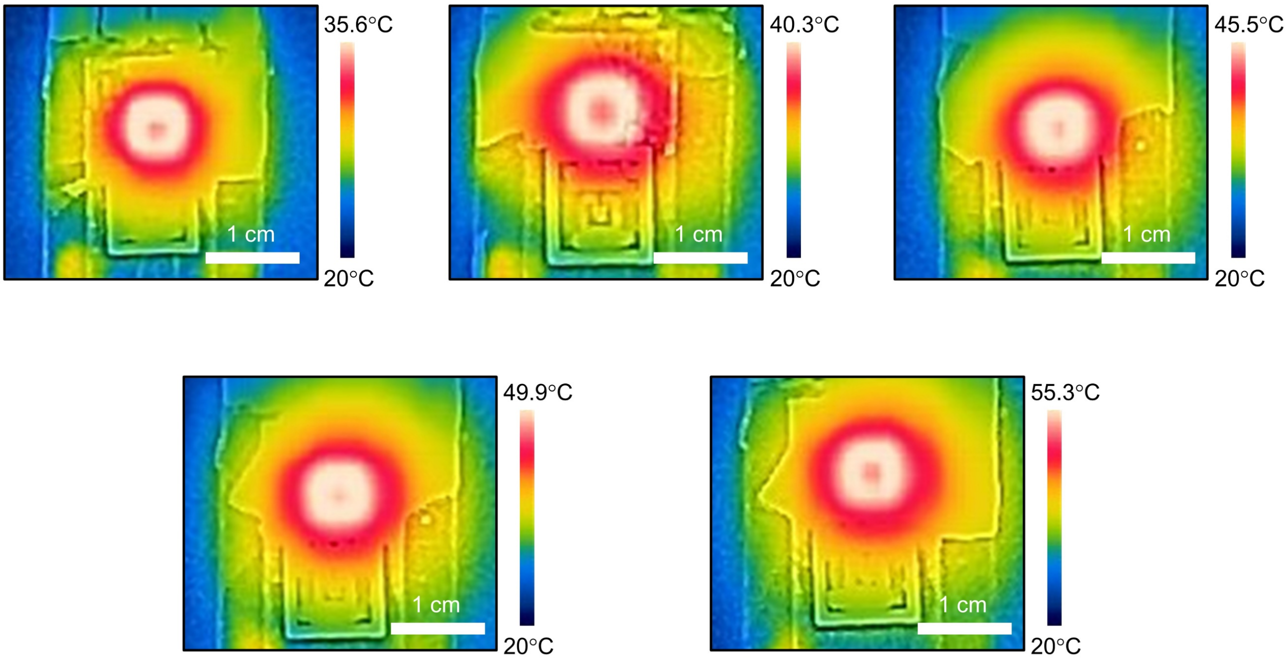

Fig. S4. Thermal distribution of the OGs insides with the target temperatures varying from 35°C to 55°C. Here, due to the fully encapsulated structure design, we remove the PET cantilever and breathing layers for exposing the inner heating parts contributed from both the copper coil and the heating electrode.

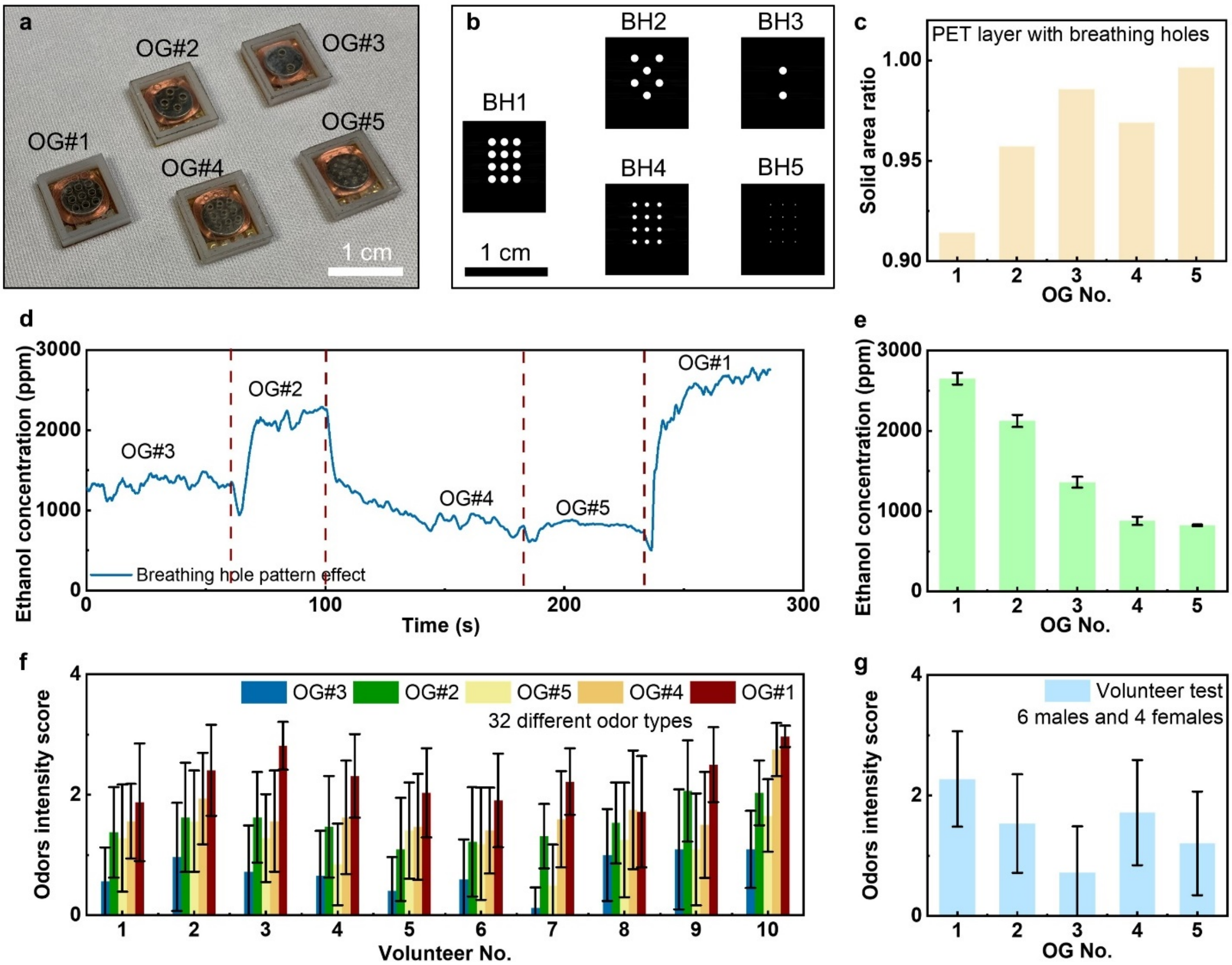

Fig. S5. Optimization of the breathing hole pattern in the top PET layer of OGs. **a** Optimical image of five OGs with different breathing hole patterns with enlarged details shown in **b**. **b** Designs of five different

breathing hole patterns in the top PET layer of OGs. **c** Solid area ratios of the top PET layer as a function of the five different breathing hole patterns. **d** Ethanol concentration generated by the five OGs with different breathing hole patterns as a function of operation time. **e** The stabilized ethanol concentration generated by OGs as a function of the breathing hole patterns. Here, it is obvious that the OG#1 with a lowest solid area ratio of the top PET layer could contribute to a highest ethanol concentration (~2649 ppm). **f, g** A volunteer test showing the odor intensity sensed by 10 volunteers as a function of the five different breathing hole patterns for 32 different odor types (same to those in Fig. 3f). Here, there are 6 males and 4 females for the volunteer test. The odor intensity scores range from 0 to 3, corresponding to four odor intensities, including none, low, middle, and high. During the volunteer test, volunteers were asked what intensity score they sensed after smelling the same odors generated by the five OGs. The error bars are presented here as the standard deviation.

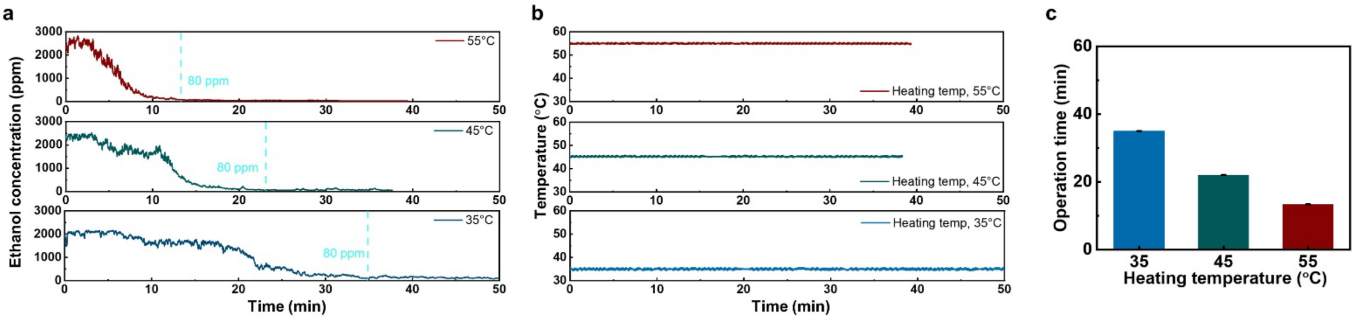

Fig. S6. a, c Ethanol concentration generated by the OGs as a function of operation time when the heating temperatures (**b**) of the OGs are targeted at 55°C, 45°C, and 35°C, respectively. The error bars are presented as the standard deviation.

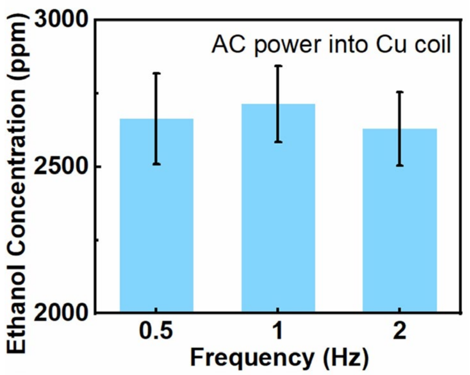

Fig. S7. Ethanol generation performance of the OGs as a function of the frequency of AC current into copper coil. The error bars are presented as the standard deviation.

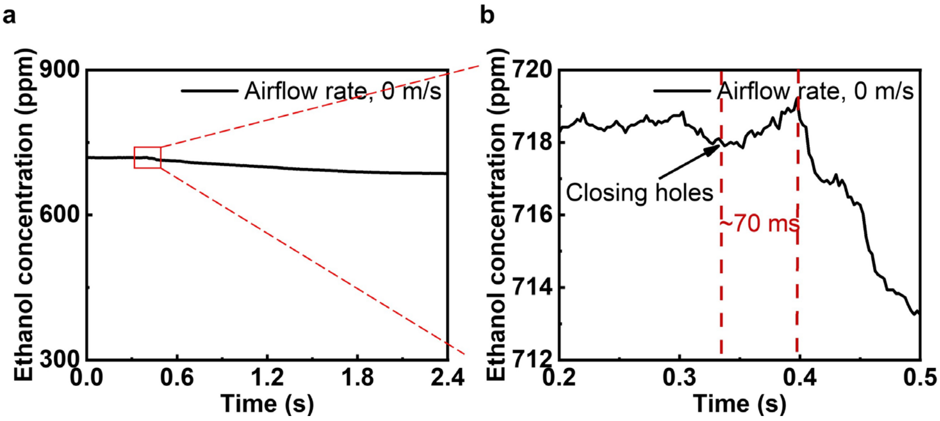

Fig. S8. Ethanol concentration response along with the operation time, during which the OG is programmed to close the breathing holes at 0.33 s with the ambient wind speed of 0 m/s.

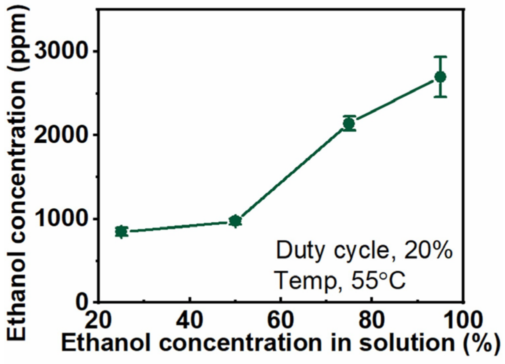

Fig. S9. Ethanol generation performance of the OGs as a function of the ethanol concentration in the solution added into the OGs. The error bars are presented as the standard deviation.

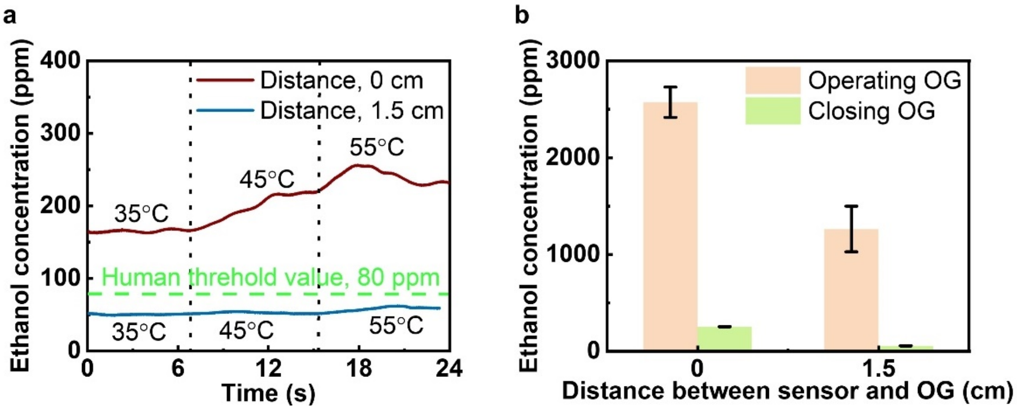

Fig. S10. Ethanol concentration generated by the OG as a function of the distance between the ethanol sensor and the breathing holes of the OG, where the OG is programmed to continuously close the breathing holes. During the test, the heating temperature of the OG is generally increased from 35°C to 55°C to investigate the heating temperature effect on the leakage of ethanol gas from the OG. The error bars are presented as the standard deviation.

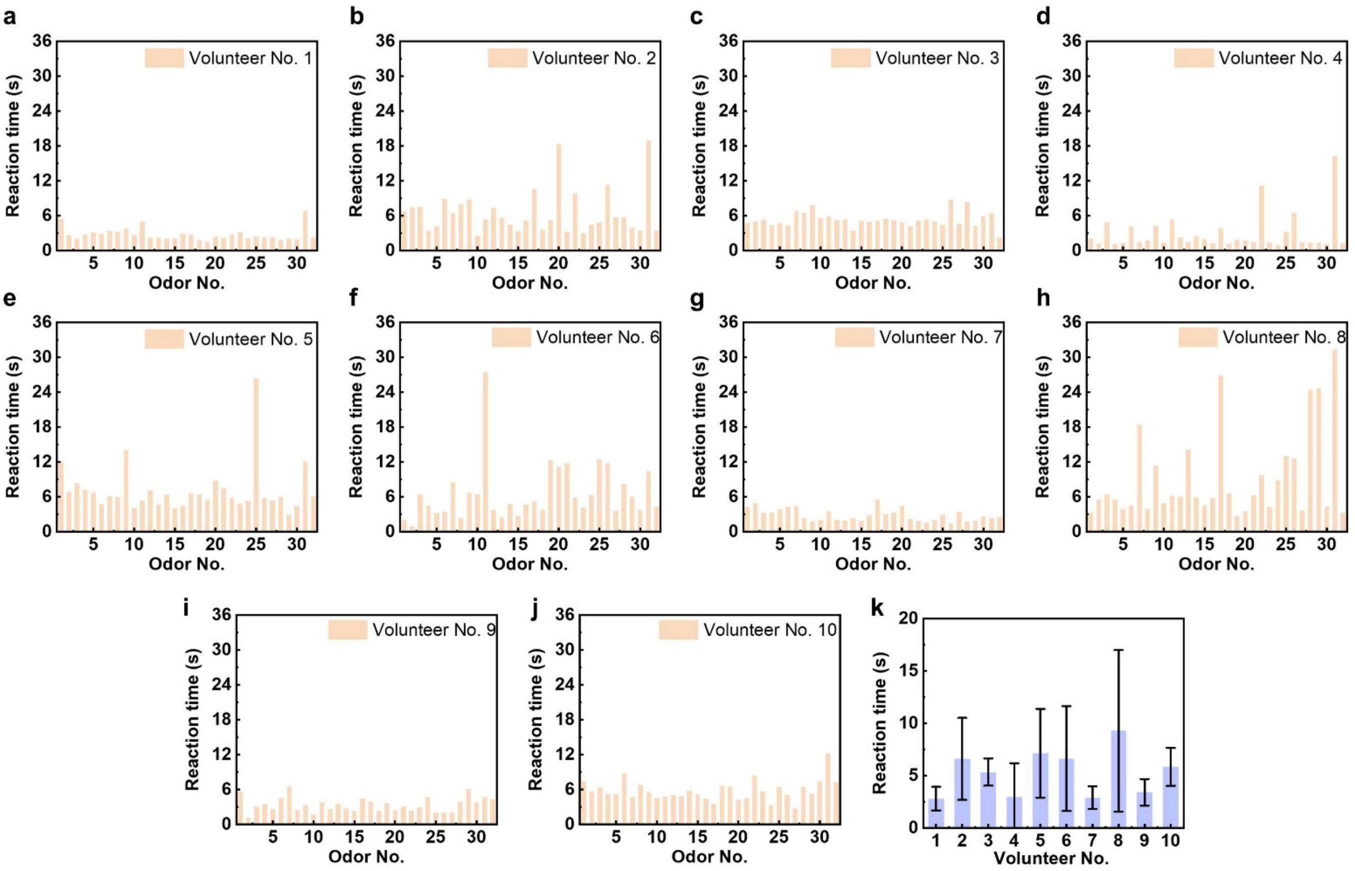

Fig. S11. A volunteer test showing that the odor reaction time of the 10 volunteers as a function of 32 different odor types (see odor details in Characteristics) at a constant distance between the OGs and the volunteers' nose of 1.5 cm. As observed in Fig. 2g, the longer reaction time to a specific odor could contribute to a higher odor concentration accumulated around the OGs, corresponding to a higher olfactory threshold value to the odor. Therefore, it is concluded that the same person may have different olfactory thresholds for different odors, and different people may also have distinguished thresholds for a same odor. The error bars are presented as the standard deviation.

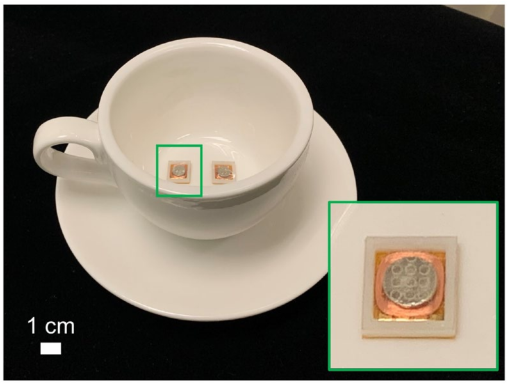

Fig. S12. Optical image of a cup with two OGs insides for generating two different drink odor types, including green tea and coffee milk.

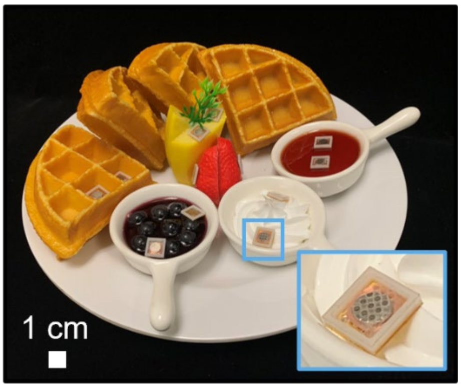

Fig. S13. Optical image of the fake pancake dish with 10 OGs integrated for generating different food odor types.

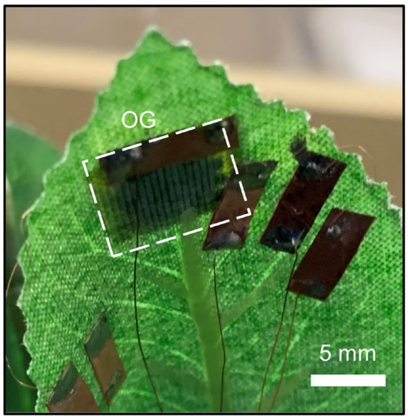

Fig. S14. Optical image of the wire connection detail at the back of OGs mounted fake leaf.

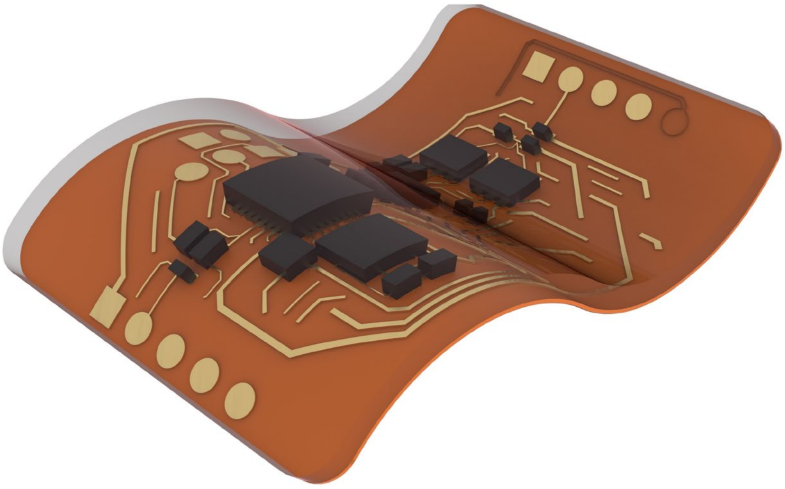

Fig. S15. Schematic diagram of the control panel for the two-channel olfaction interface.

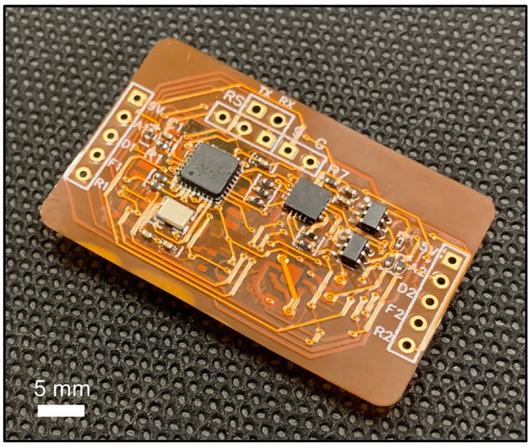

Fig. S16. Optical image of the backside control panel without encapsulation layers.

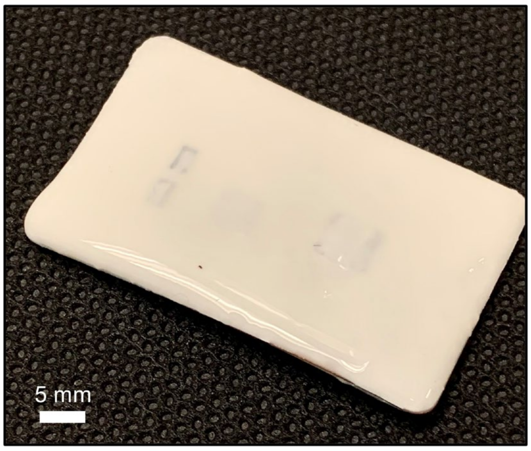

Fig. S17. Optical image of the backside control panel with colored encapsulation layers.

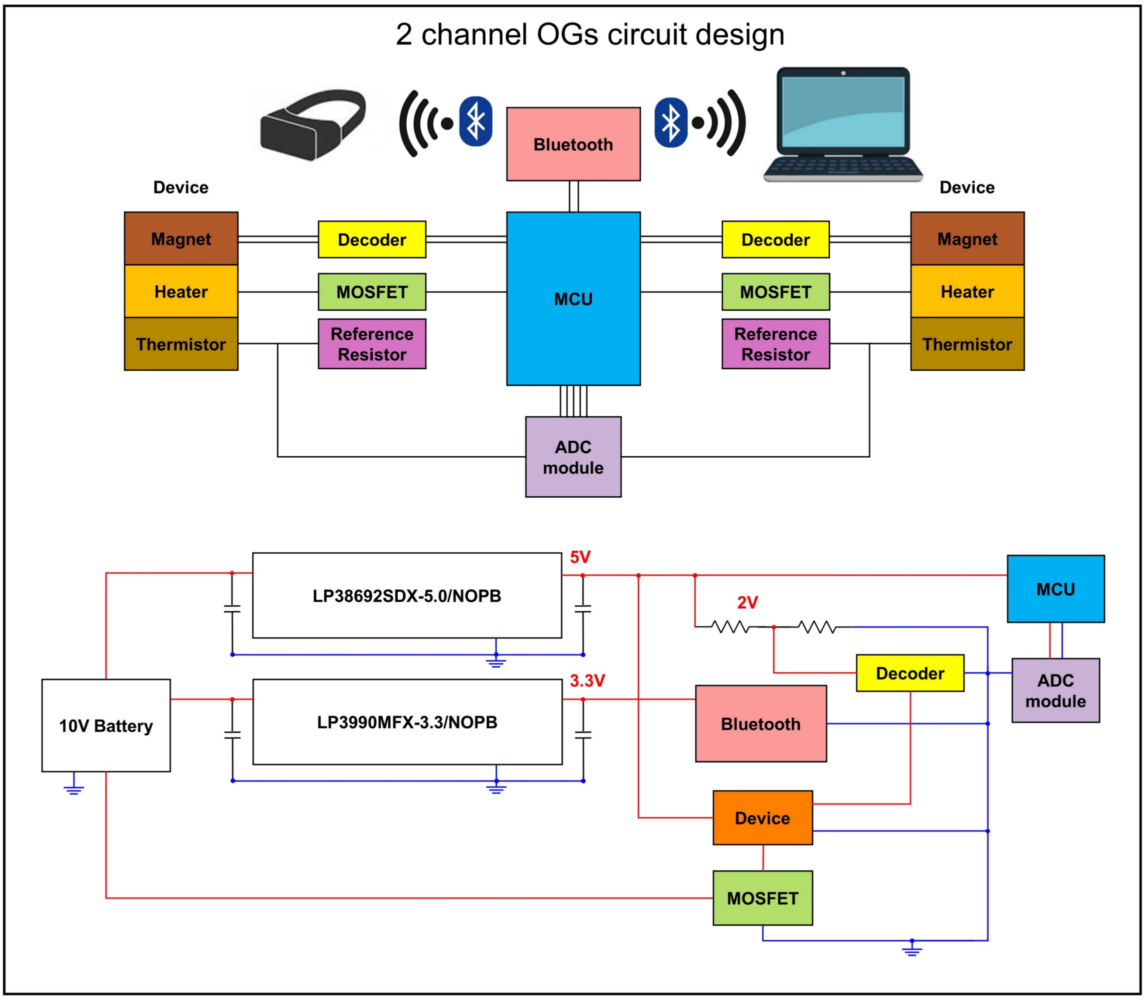

Fig. S18. Detailed circuit design of the control panel for the two-channel olfaction interface.

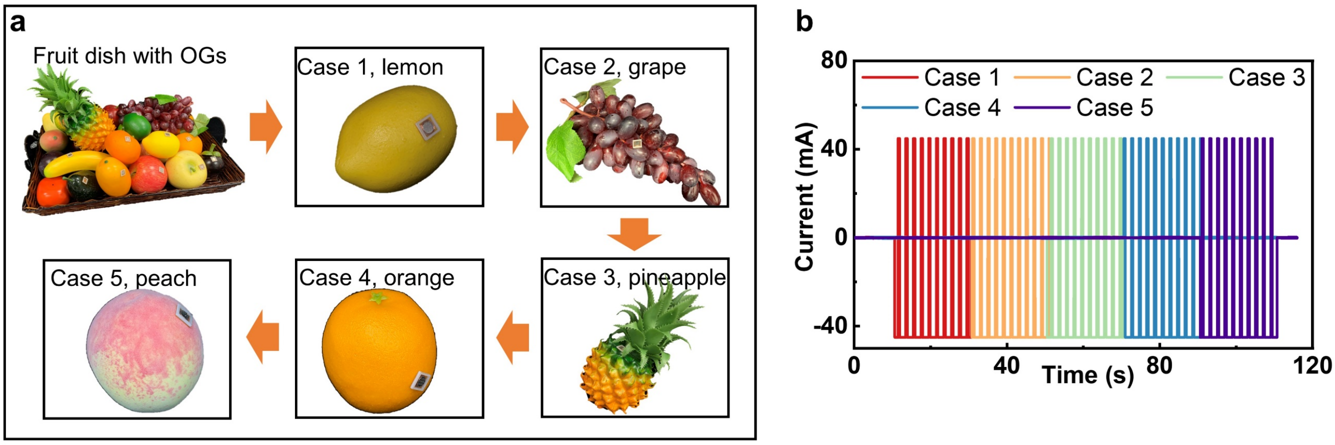

Fig. S19. A fake fruit basket with multiple OGs integrated, including but not limited to lemon, grape, pineapple, orange, and peach. By programming the olfaction interface, the five OGs insides the fruit basket could operate one by one with odor types varying from lemon to grape, to pineapple, to orange, and to peach.

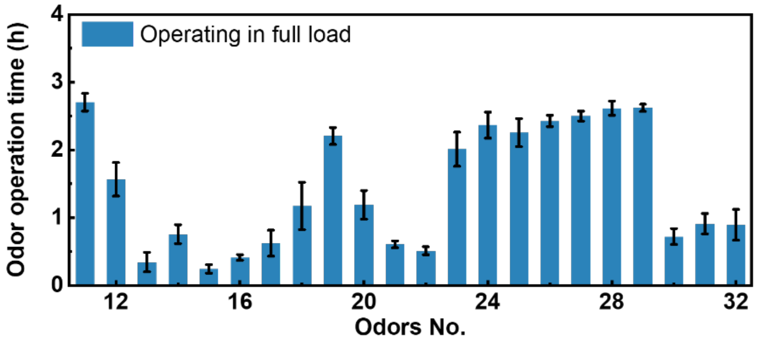

Fig. S20. Full-load odor operation time as a function of odor No. ranging from Odor No. 11 to No. 32. Here, the error bars are presented as the standard deviation, and the test was repeated 3 times by testing 3 volunteers.

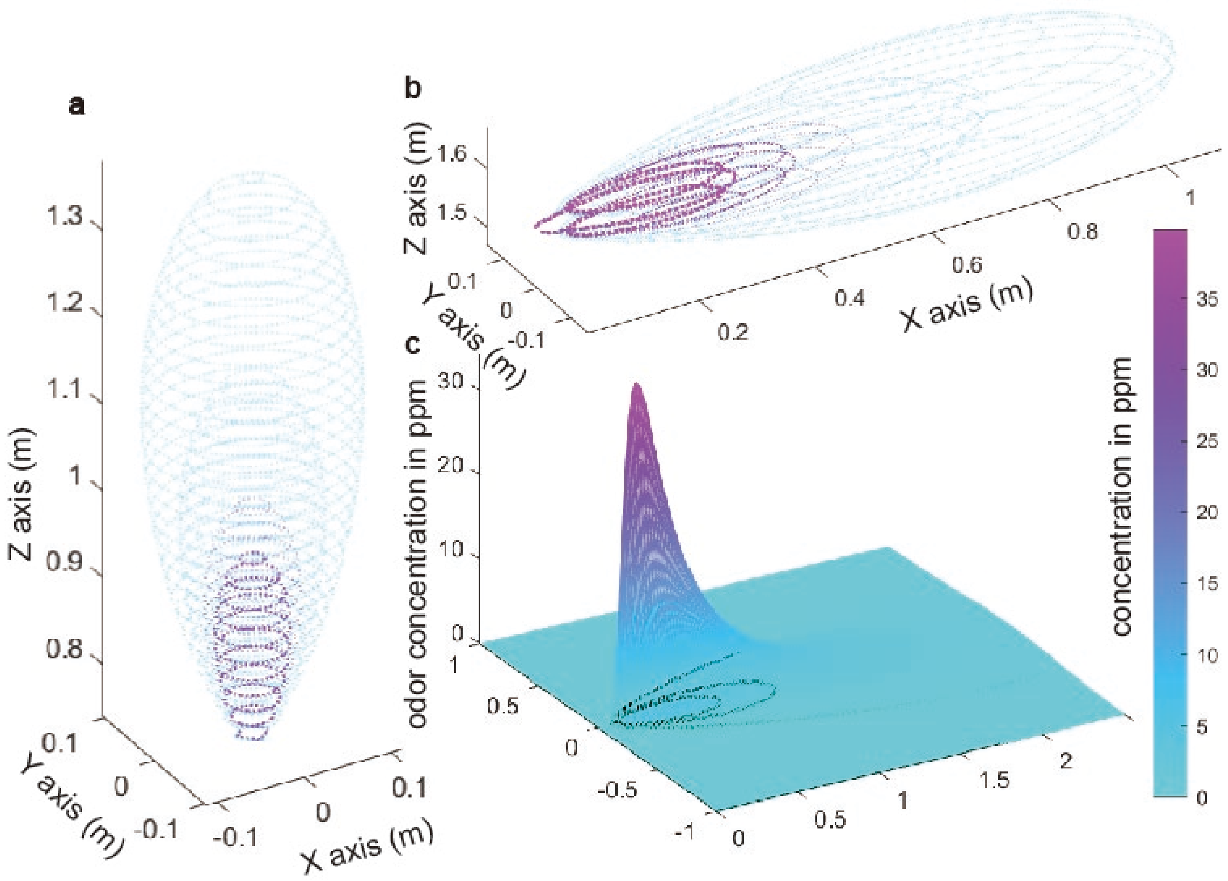

Fig. S21. Simulated plume structure of OS 4 with vertical convective airflow (**a**) and OS 2 with horizontal environmental airflow (**b**). **c** a slicing of **b** at its central height.

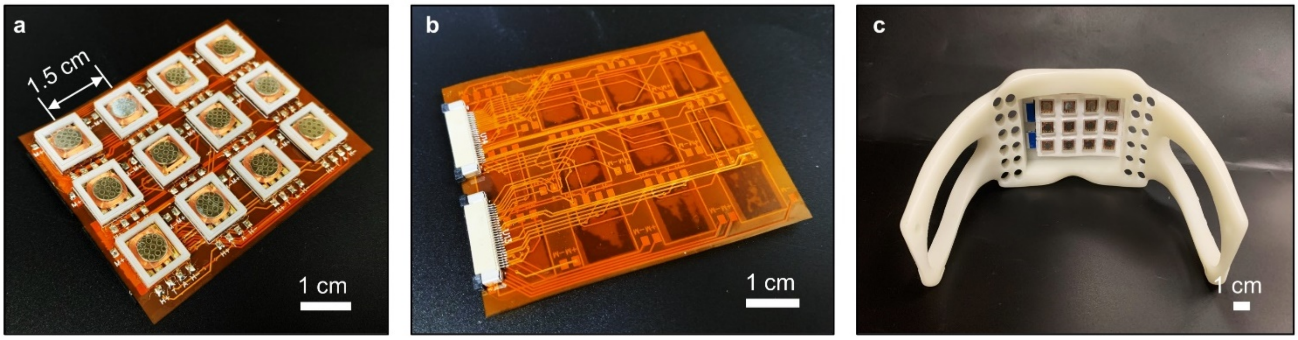

Fig. S22. a, b Optical images of the 4 × 3 OGs array mounted onto a self-designed FPCB with the top and bottom views. c Optical image of the face-mask olfactory interface based on the 4 × 3 OGs array.

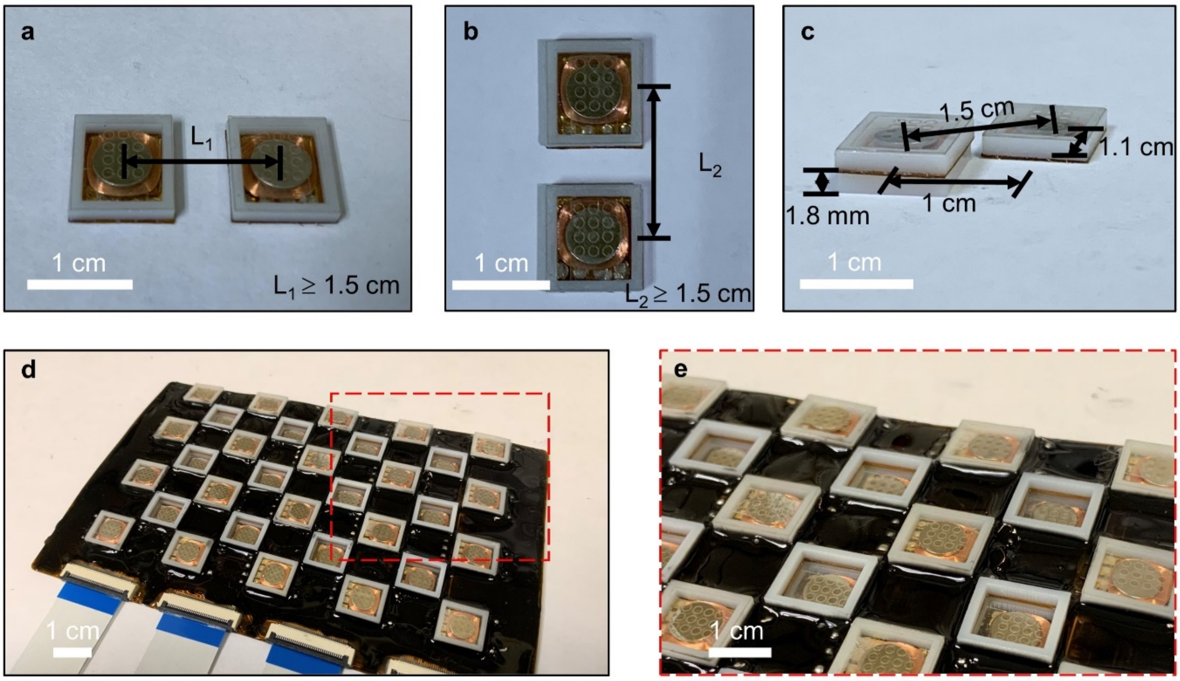

Fig. S23. a, b, c Optical images of the three two-OG layouts. **d, e** Optical images of the 32-channel olfaction interface with enlarged details for presenting the OGs layout method.

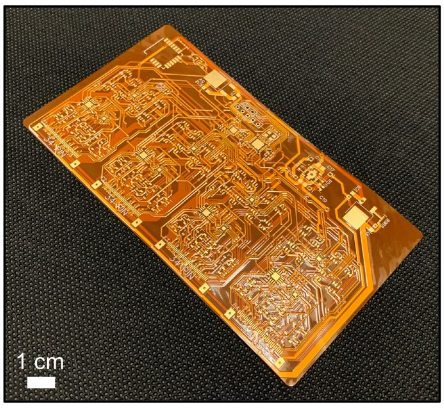

Fig. S24. Optical image of the control panel FPCB for the 32-channel olfaction interface.

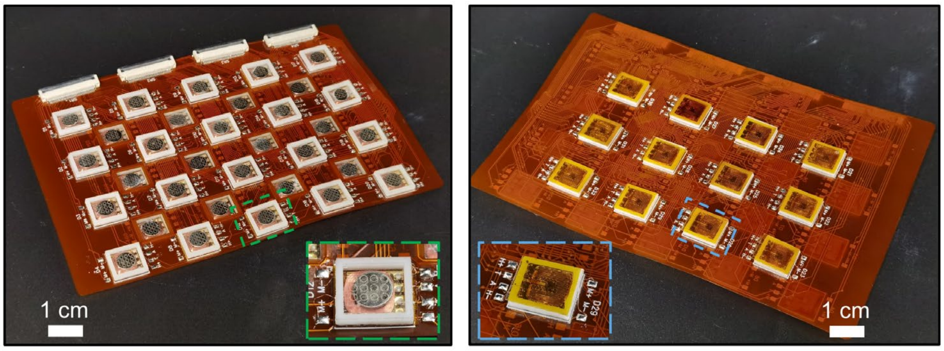

Fig. S25. Optical images of the 32-channel olfaction interface without the encapsulation layers.

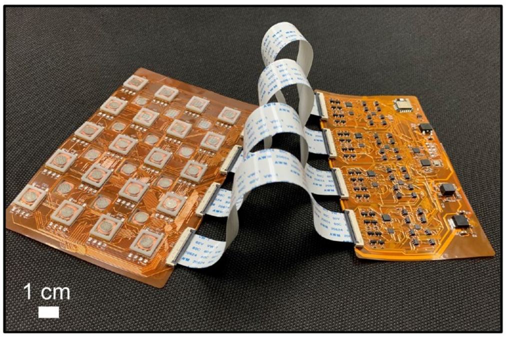

Fig. S26. Optical images of whole the 32-channel olfaction system without encapsulation layers.

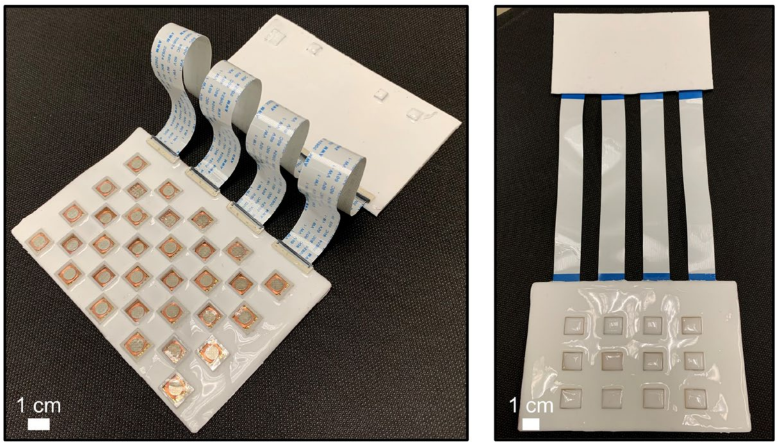

Fig. S27. Optical images of whole the 32-channel olfaction system with encapsulation layers.

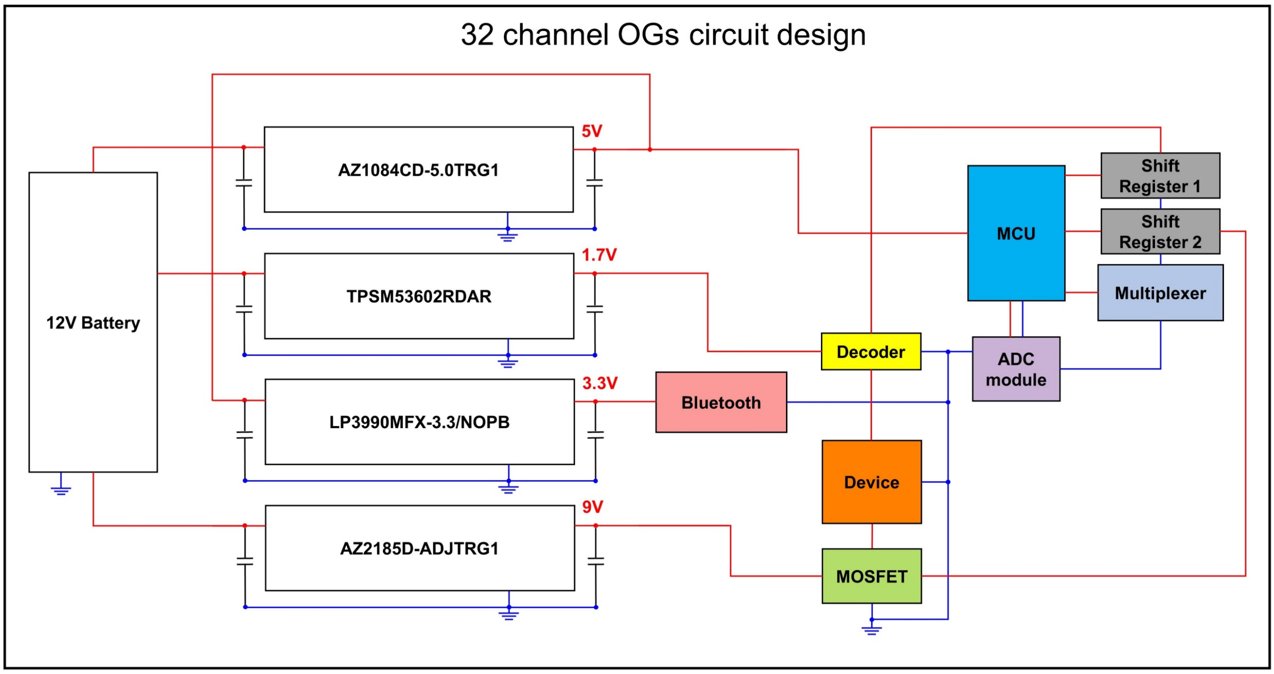

Fig. S28. Detailed circuit design of the control panel for the 32-channel olfaction interface.

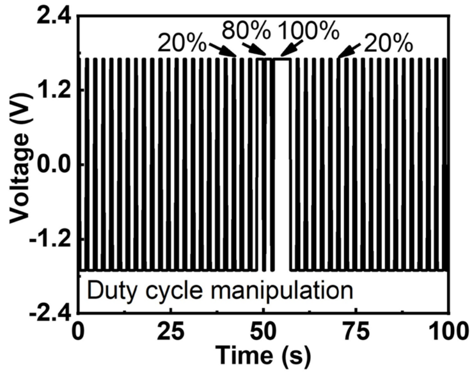

Fig. S29. Duty cycle variation of the voltage output into an OG, manipulated by the control panel of the 32-channel olfaction system.

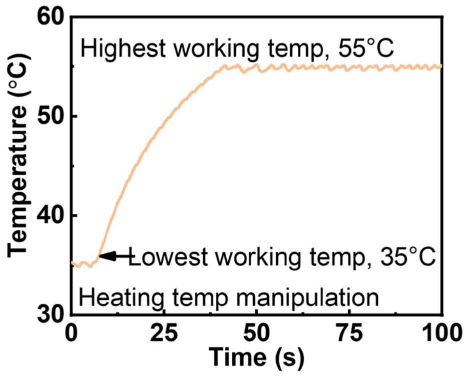

Fig. S30. Heating temperature of an OG varies between 35 and 55°C, manipulated by the control panel of the 32-channel olfaction system.

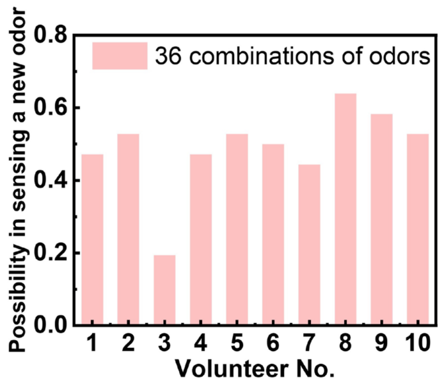

Fig. S31. A volunteer test demonstrating the possibility for volunteers in sensing a new odor when two different odors are generated by the 32-channel olfactory interface at one time. Here, we randomly selected 9 different odors to create 36 (C_9^2) different odor combinations for the volunteer test, including strawberry, cake, ginger, lemon, rice, tobacco, green tea, lavender, and vanilla.

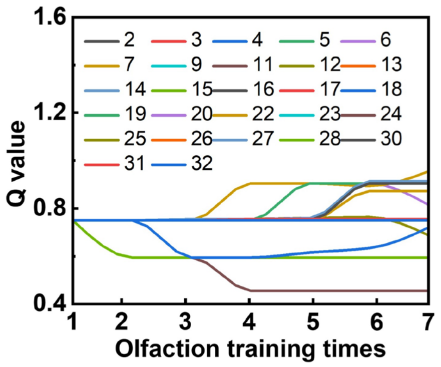

Fig. S32. 32-odor Q values of one volunteer as a function of olfaction training times in the LRF training method.

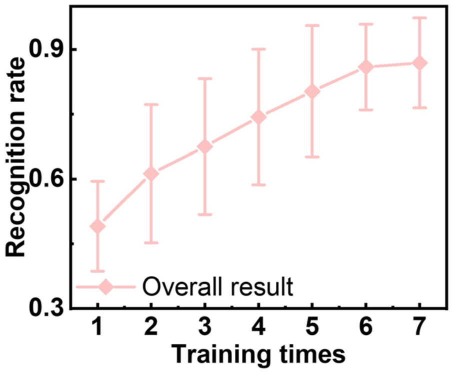

Fig. S33. Average recognition rate from 10 volunteers as a function of training times in the LRF method. The error bars are presented as the standard deviation.

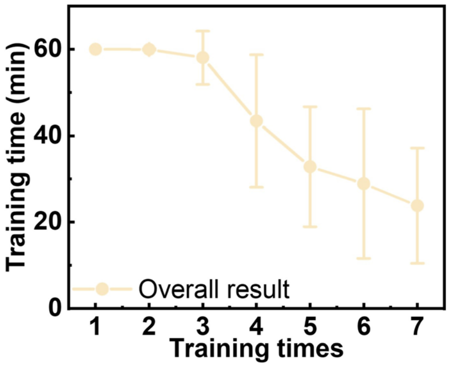

Fig. S34. Average training time from 10 volunteers as a function of training times in the LRF method. The error bars are presented as the standard deviation.

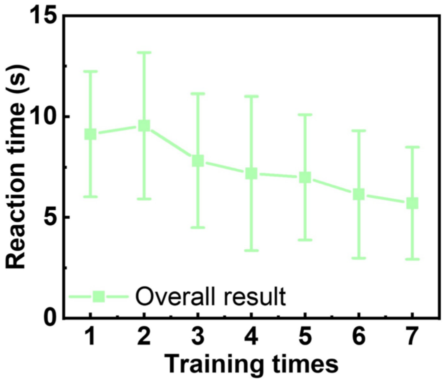

Fig. S35. Average reaction time from 10 volunteers as a function of training times in the LRF method. The error bars are presented as the standard deviation.

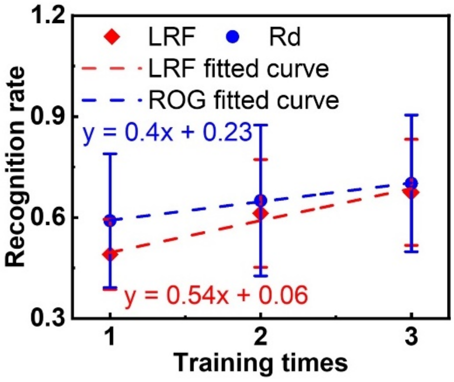

Fig. S36. Comparison between the two different training methods, LRF and ROG, in enhancing volunteers' odor recognition rate. The error bars are presented as the standard deviation.

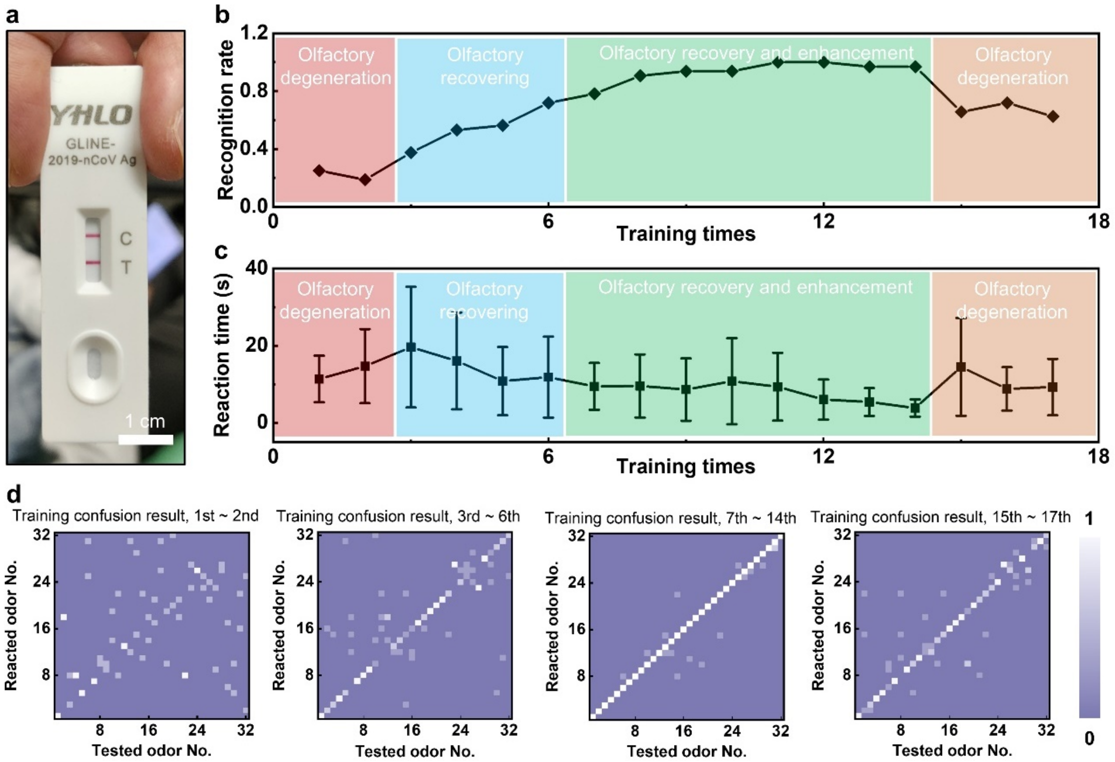

Fig. S37. A typical demonstration of the 32-channel olfaction system in recovering patients' olfaction capability whose olfaction degeneration is caused by COVID-19. **a** Optical image of the patient's COVID-19 test result showing the positive result at the first training day before starting training. **b, c** Recognition

rate and response time as a function of training times in the LRF training method. Here, from the 1st to 14th training, the training frequency is fixed at 1 time per day. From the 15th to the 17th training, the frequency is extended to 1 time per three weeks for investigating olfaction training frequency effect on the patient's olfaction capability. **d** Training confusion results as a function of training times. The error bars are presented as the standard deviation.

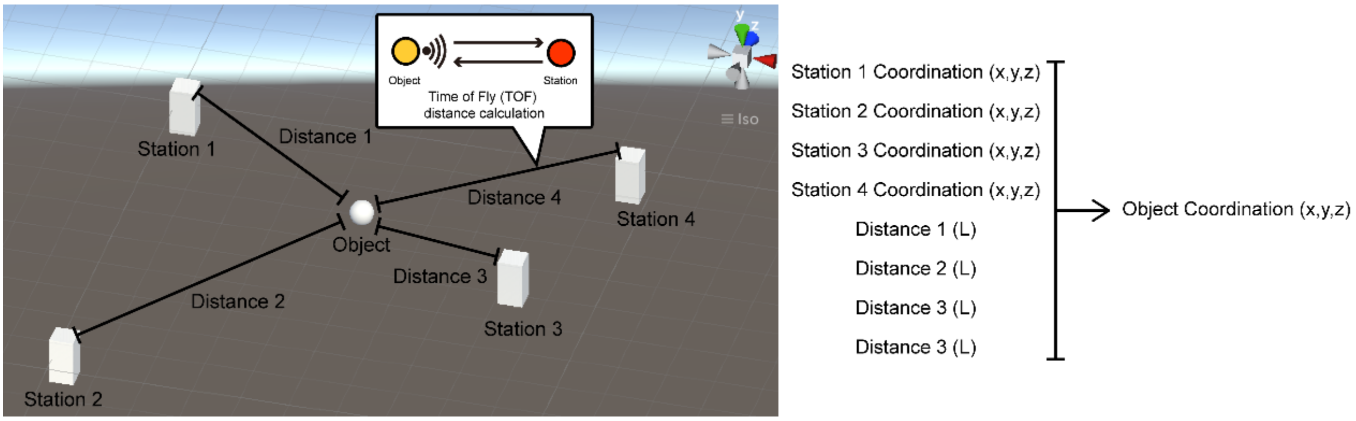

Fig. S38. Working principle of positioning system
