# Supplementary figures and images for: Intelligent wearable olfactory interface for latency-free mixed reality and fast olfactory enhancement
Source: Nat Commun. 2024 May 25;15:4474. doi: 10.1038/s41467-024-48884-z (PMC11128017; doi:10.1038/s41467-024-48884-z)

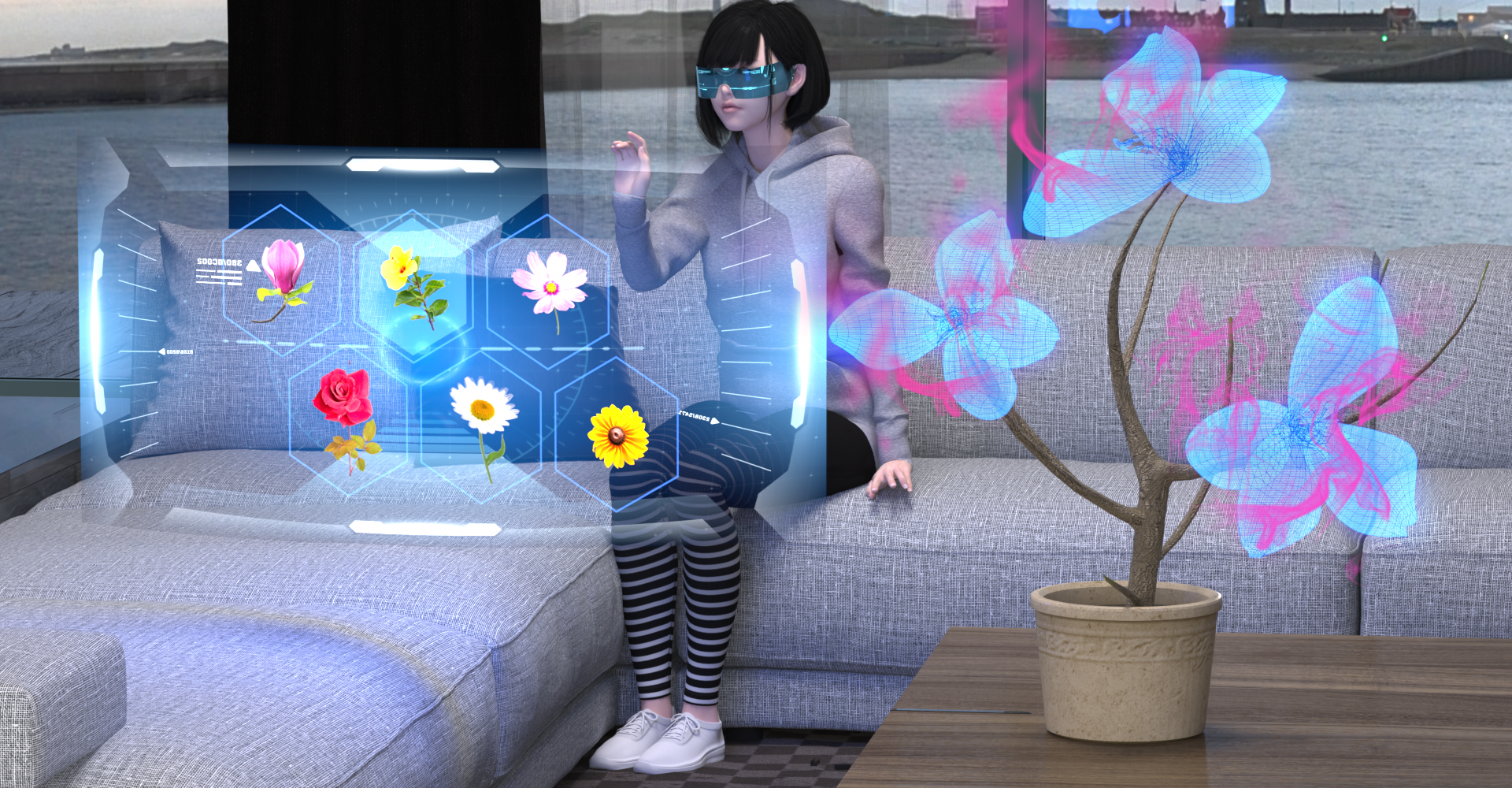

Supplement: Supplementary file 8 — Additional information on figures [file 41467_2024_48884_MOESM8_ESM.zip › Figure materials/Fig. 1g_upper left corner.tif]

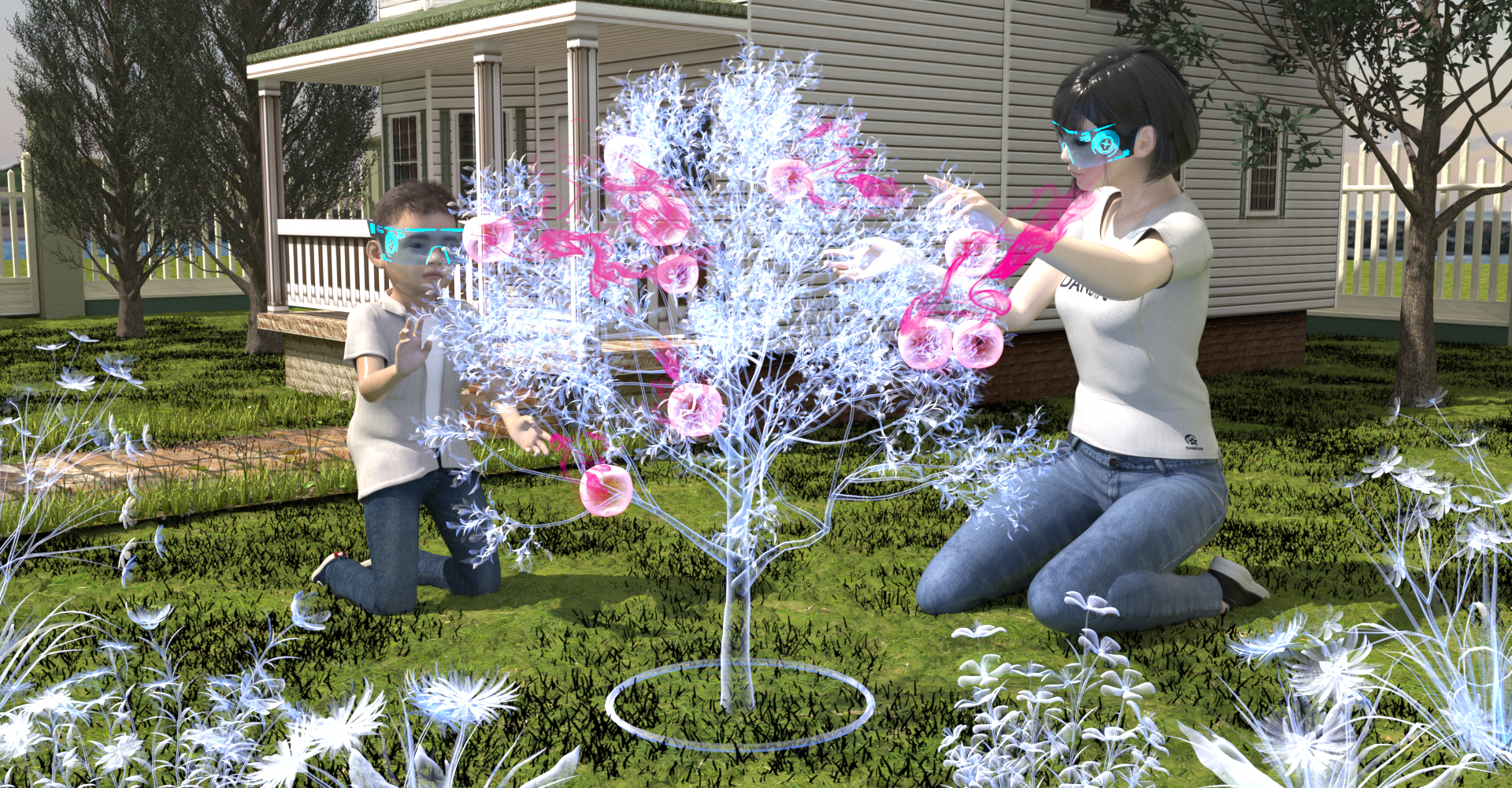

Supplement: Supplementary file 8 — Additional information on figures [file 41467_2024_48884_MOESM8_ESM.zip › Figure materials/materials for Fig. 1g_lower left corner2.tif]

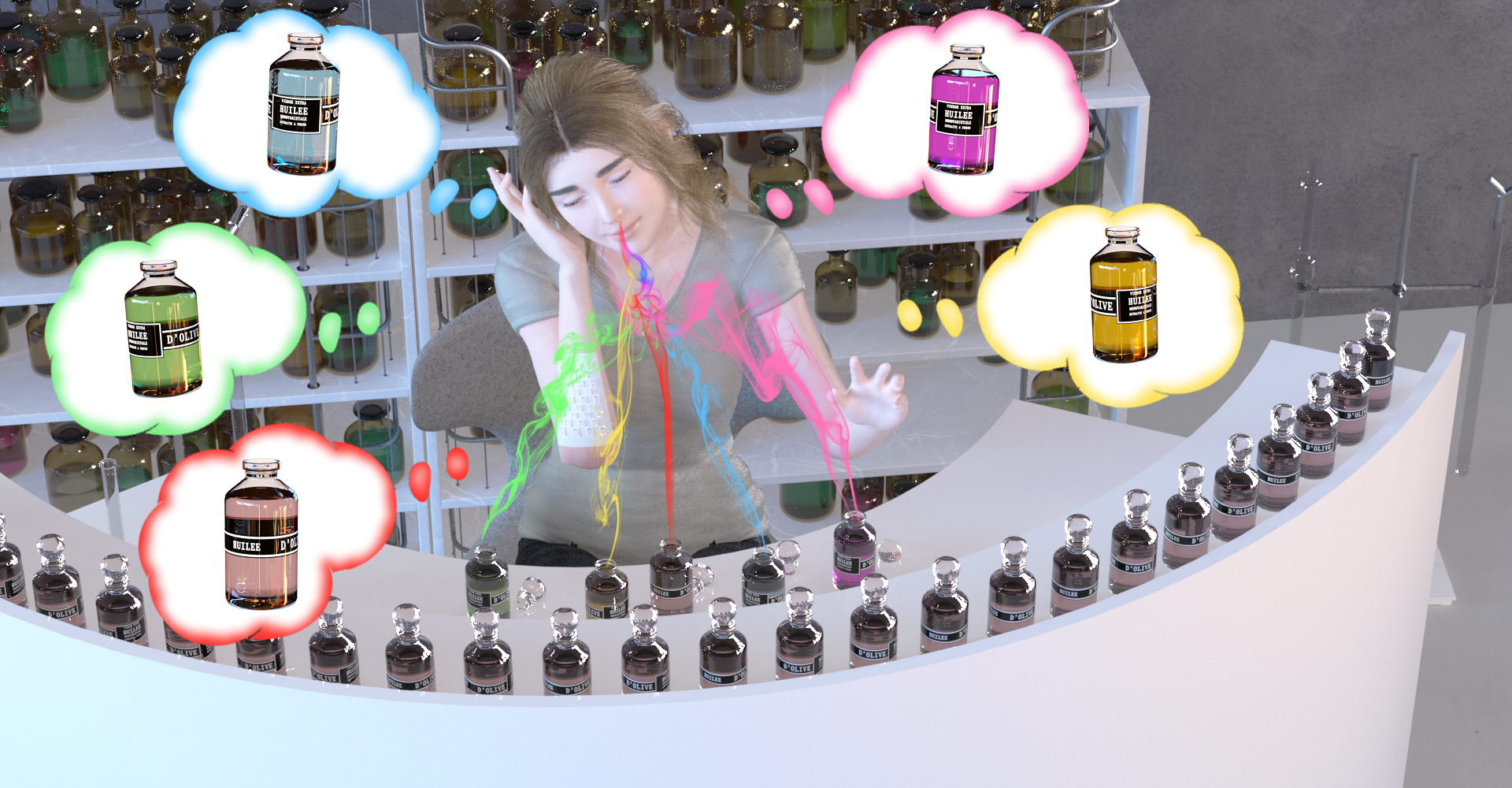

Supplement: Supplementary file 8 — Additional information on figures [file 41467_2024_48884_MOESM8_ESM.zip › Figure materials/materials for Fig. 1g_lower right corner.tif]

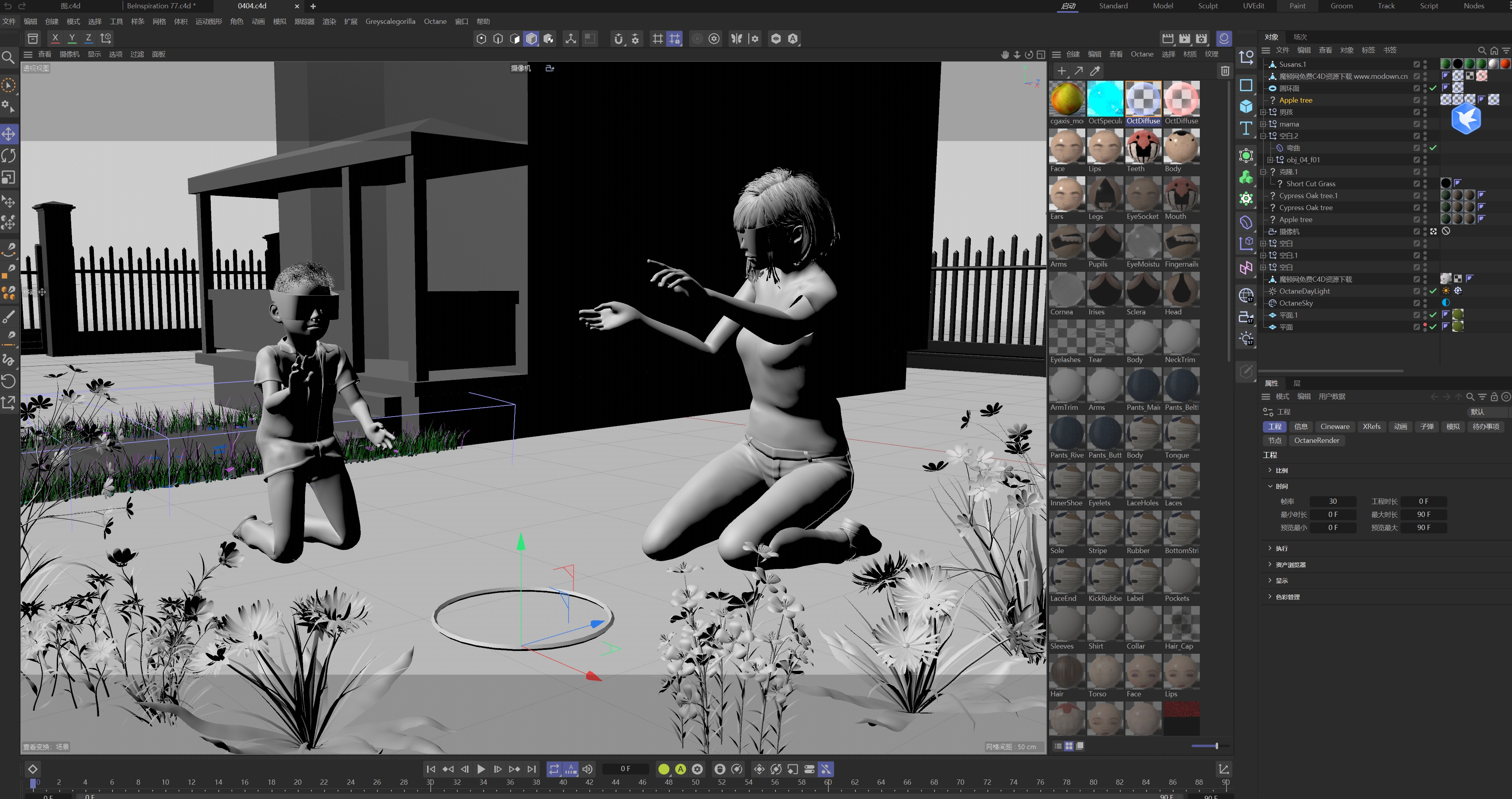

Supplement: Supplementary file 8 — Additional information on figures [file 41467_2024_48884_MOESM8_ESM.zip › Figure materials/optical images/Materials for Fig. 1g.jpg]

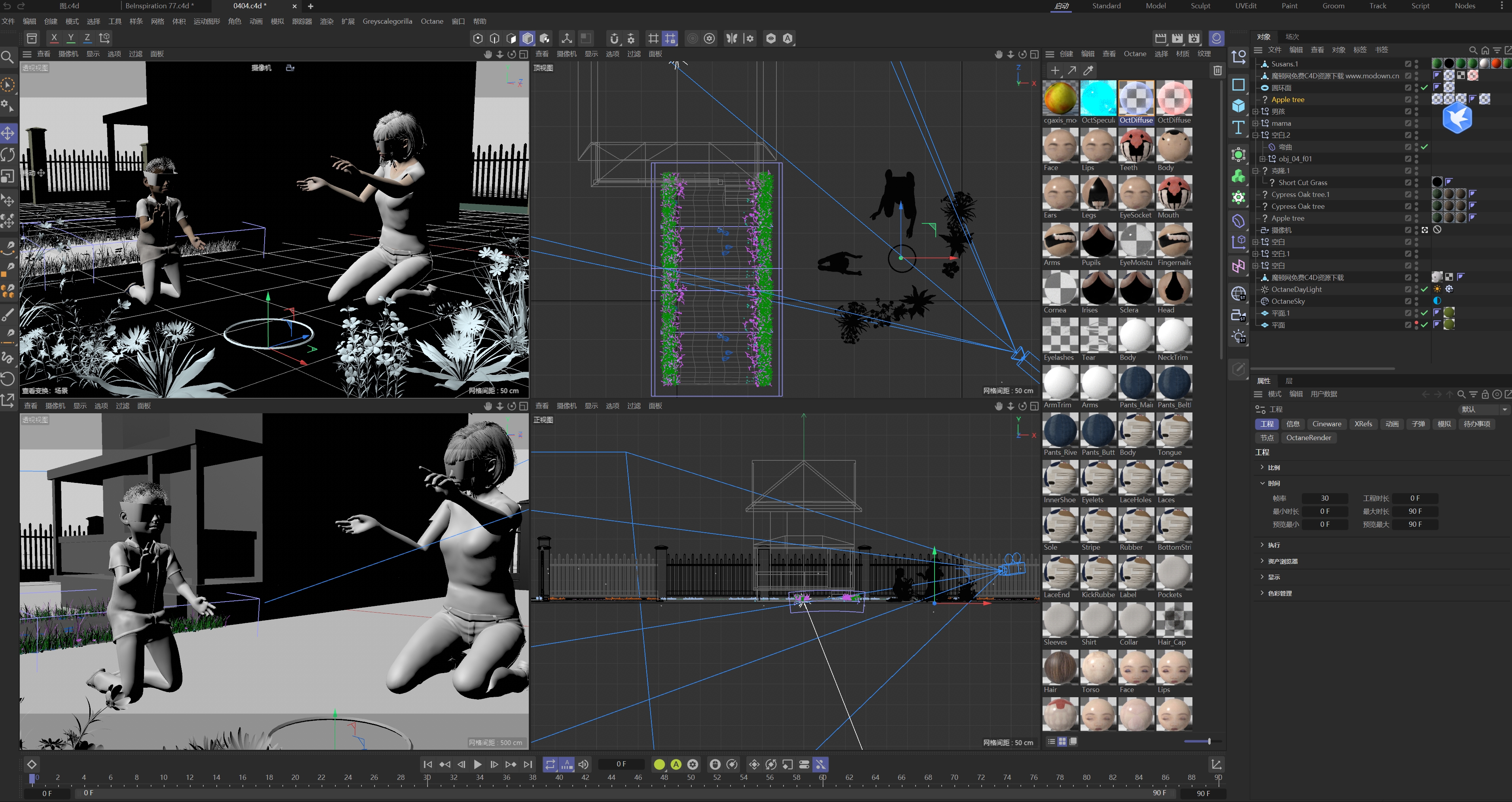

Supplement: Supplementary file 8 — Additional information on figures [file 41467_2024_48884_MOESM8_ESM.zip › Figure materials/optical images/materials for Fig. 1g_lower left corner1.jpg]

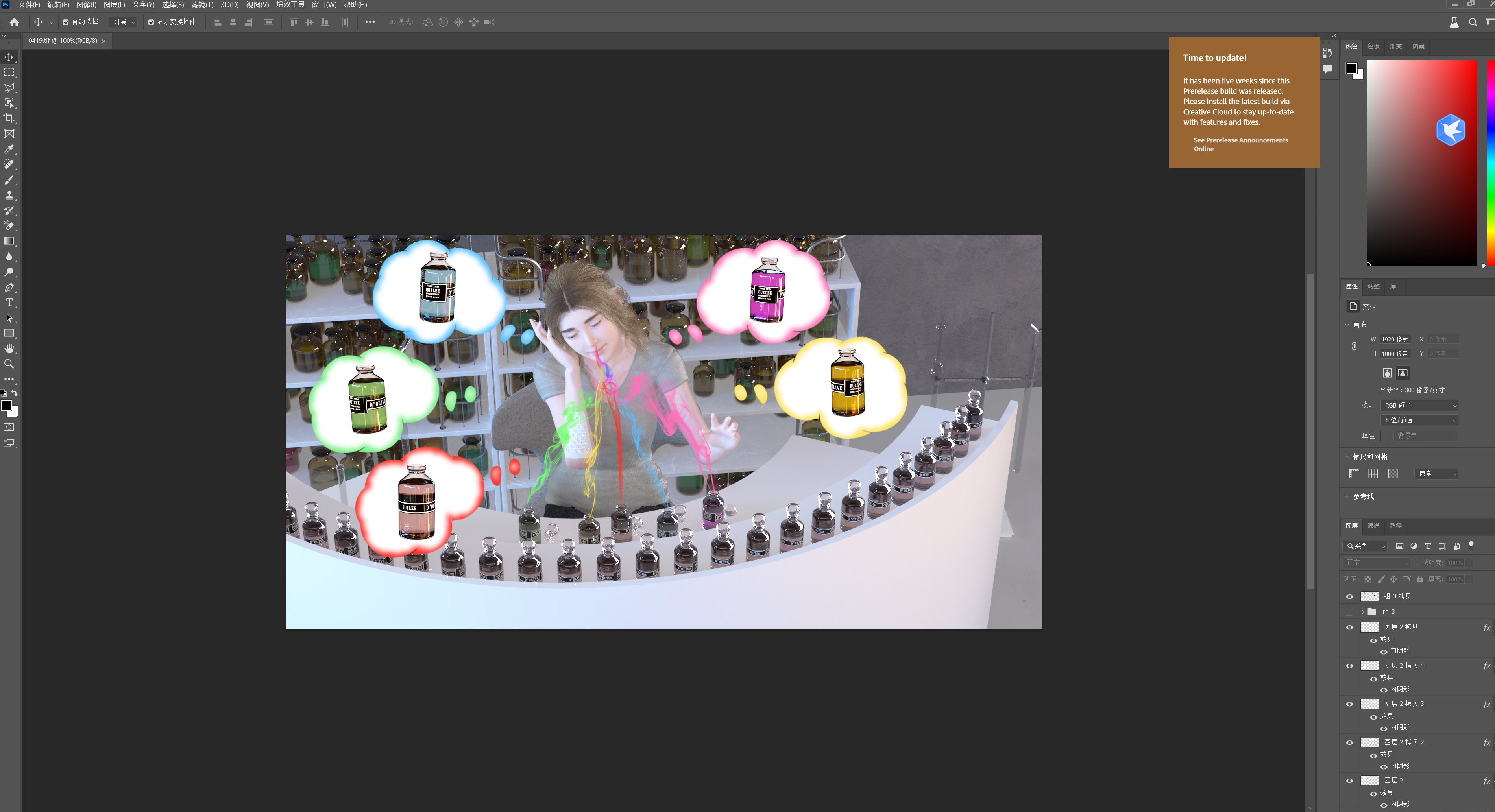

Supplement: Supplementary file 8 — Additional information on figures [file 41467_2024_48884_MOESM8_ESM.zip › Figure materials/optical images/materials for Fig. 1g_lower right corner1.jpg]

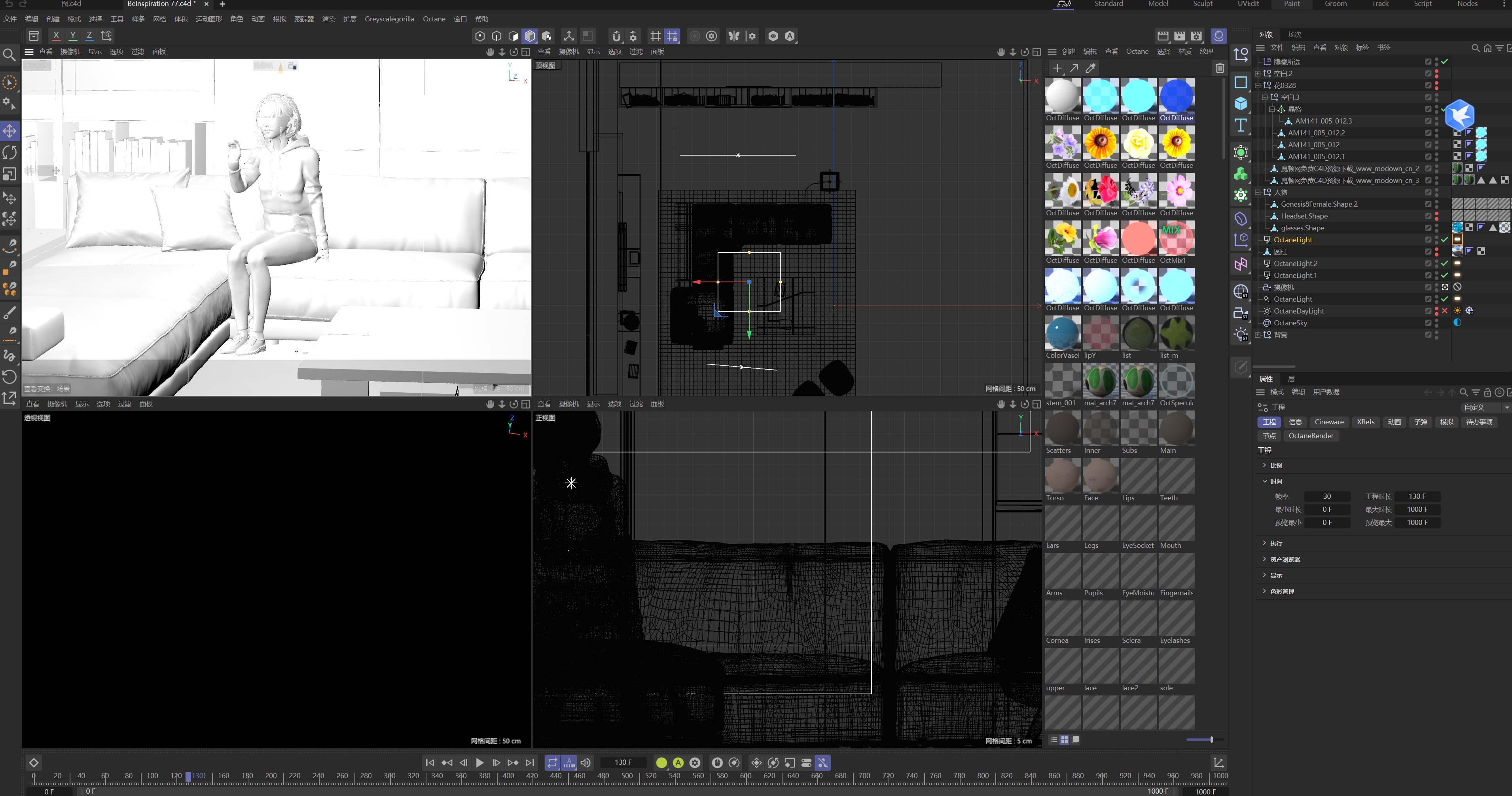

Supplement: Supplementary file 8 — Additional information on figures [file 41467_2024_48884_MOESM8_ESM.zip › Figure materials/optical images/Materials for Fig. 1g_upper left corner.jpg]

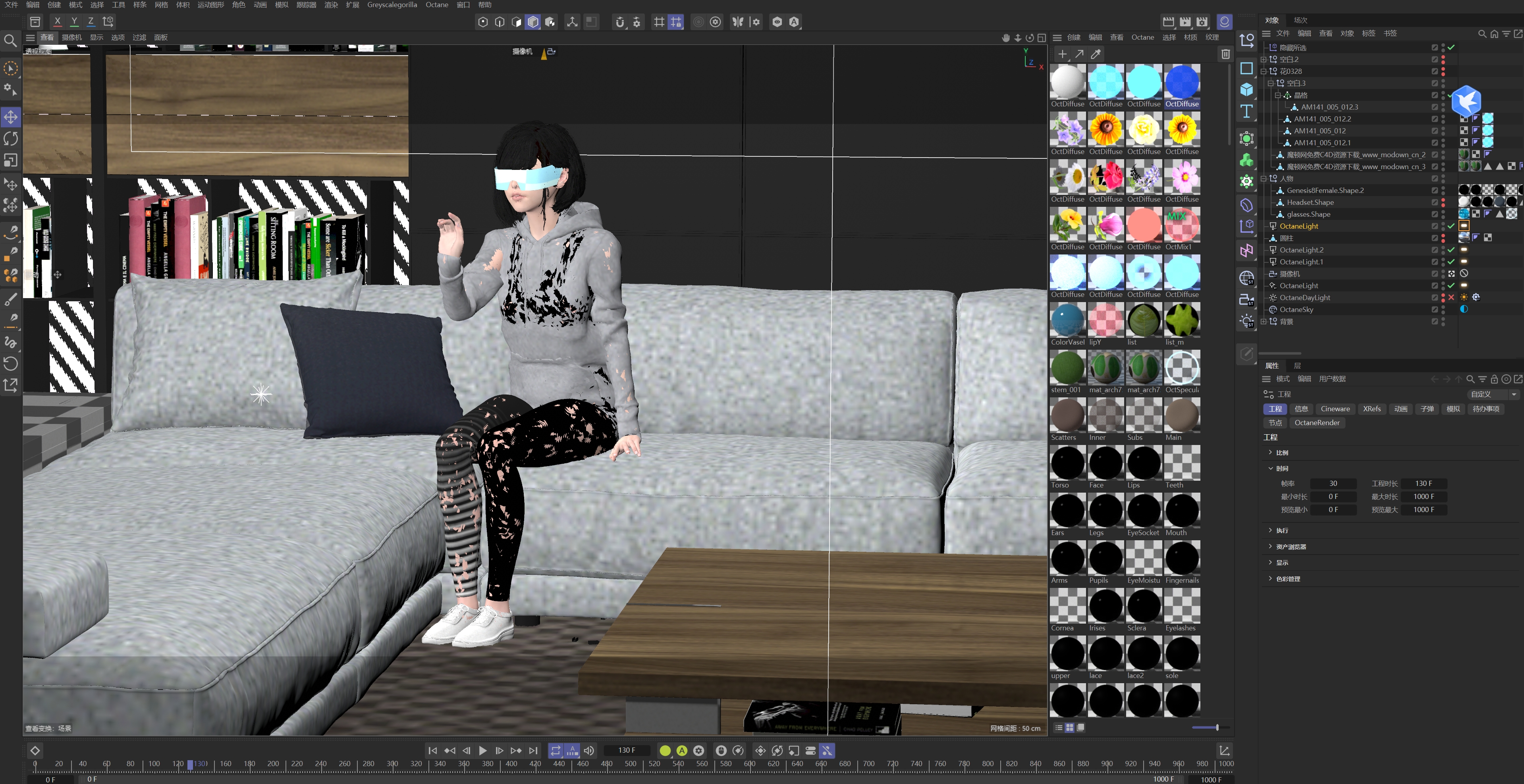

Supplement: Supplementary file 8 — Additional information on figures [file 41467_2024_48884_MOESM8_ESM.zip › Figure materials/optical images/materials for Fig. 1g_upper left corner2.jpg]

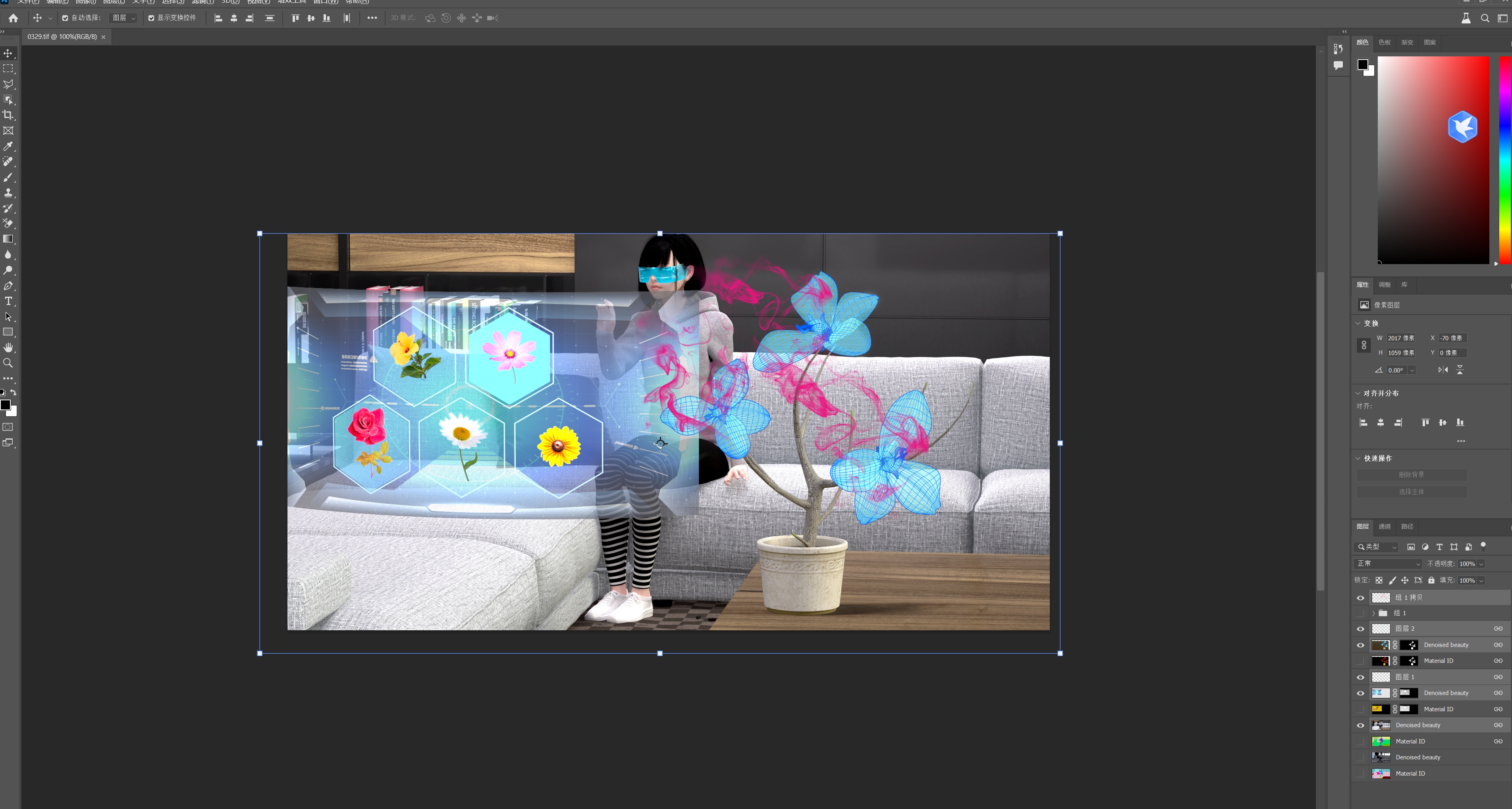

Supplement: Supplementary file 8 — Additional information on figures [file 41467_2024_48884_MOESM8_ESM.zip › Figure materials/optical images/materials for Fig. 1g_upper left corner3.jpg]

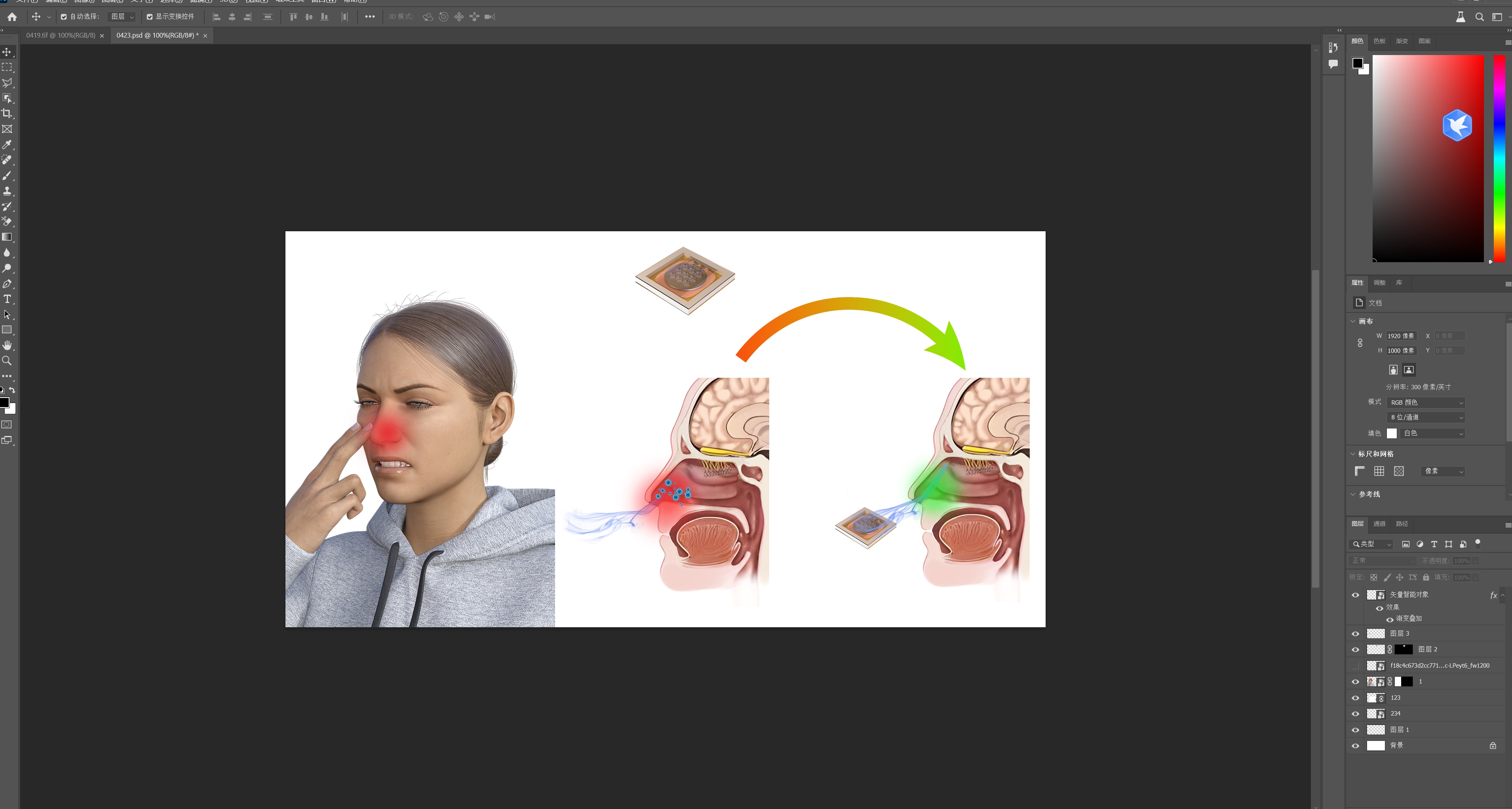

Supplement: Supplementary file 8 — Additional information on figures [file 41467_2024_48884_MOESM8_ESM.zip › Figure materials/optical images/materials for Fig. 1g_upper left corner4.jpg]

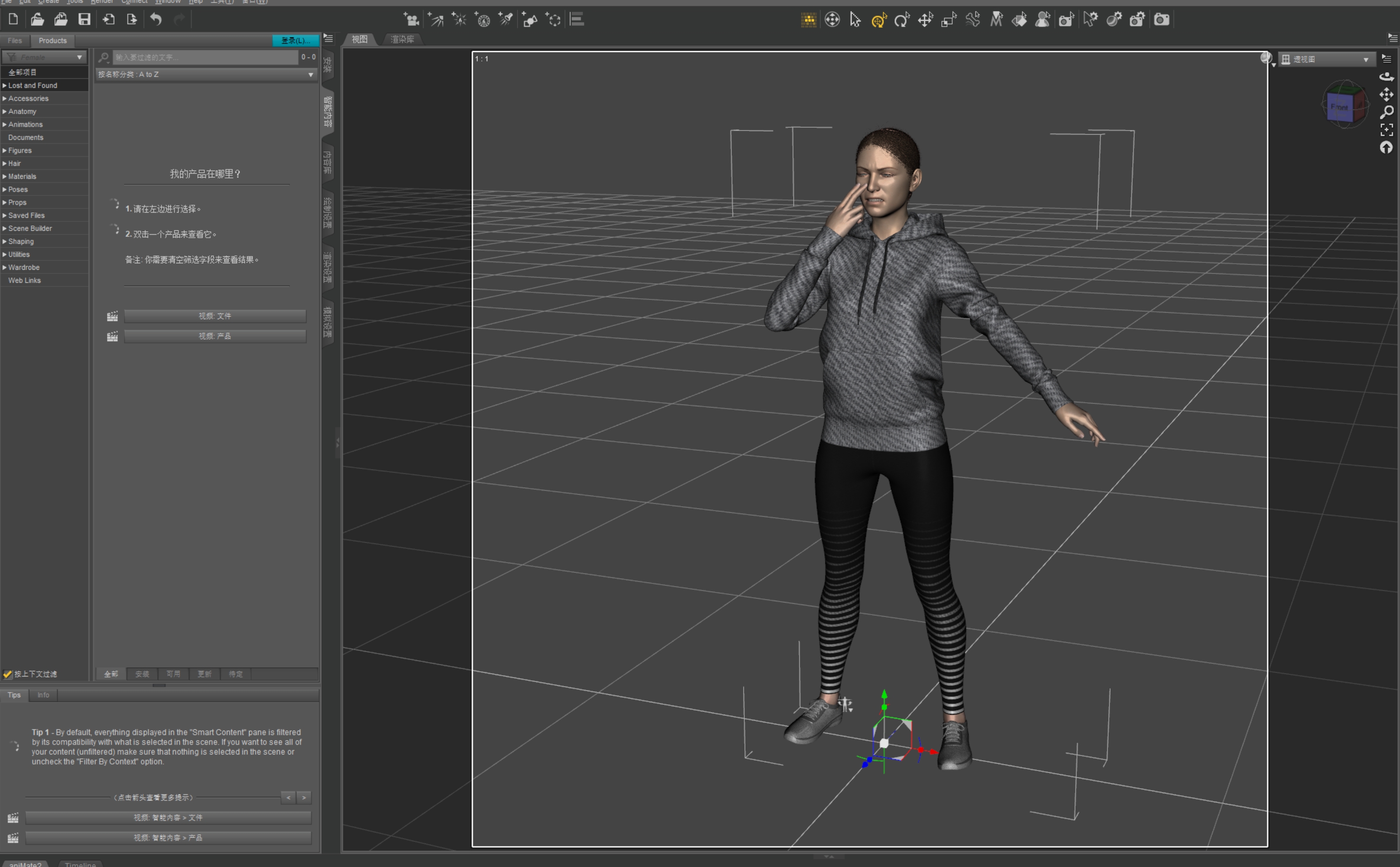

Supplement: Supplementary file 8 — Additional information on figures [file 41467_2024_48884_MOESM8_ESM.zip › Figure materials/optical images/materials for Fig. 1g_upper right corner1.jpg]

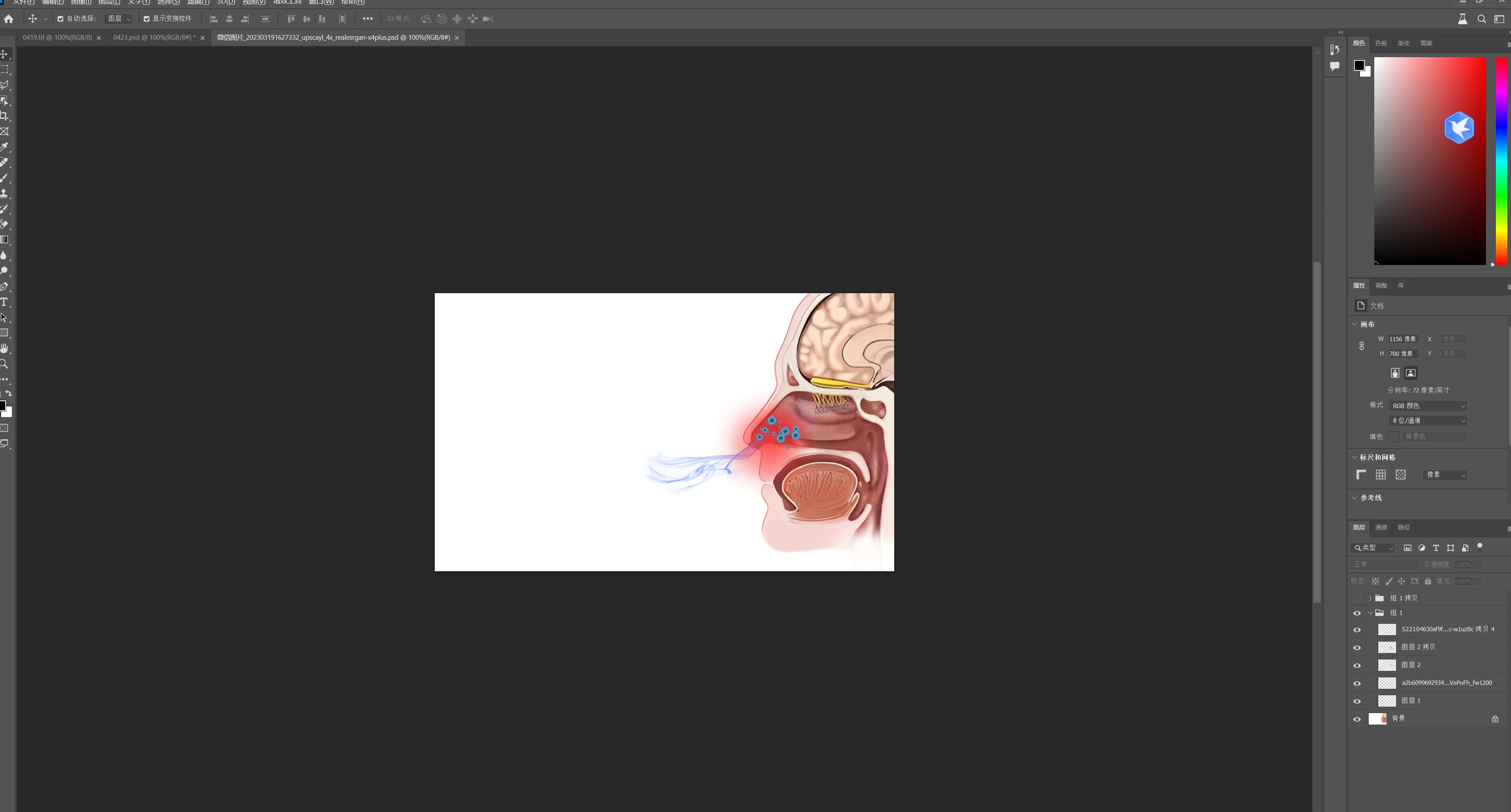

Supplement: Supplementary file 8 — Additional information on figures [file 41467_2024_48884_MOESM8_ESM.zip › Figure materials/optical images/materials for Fig. 1g_upper right corner2.jpg]

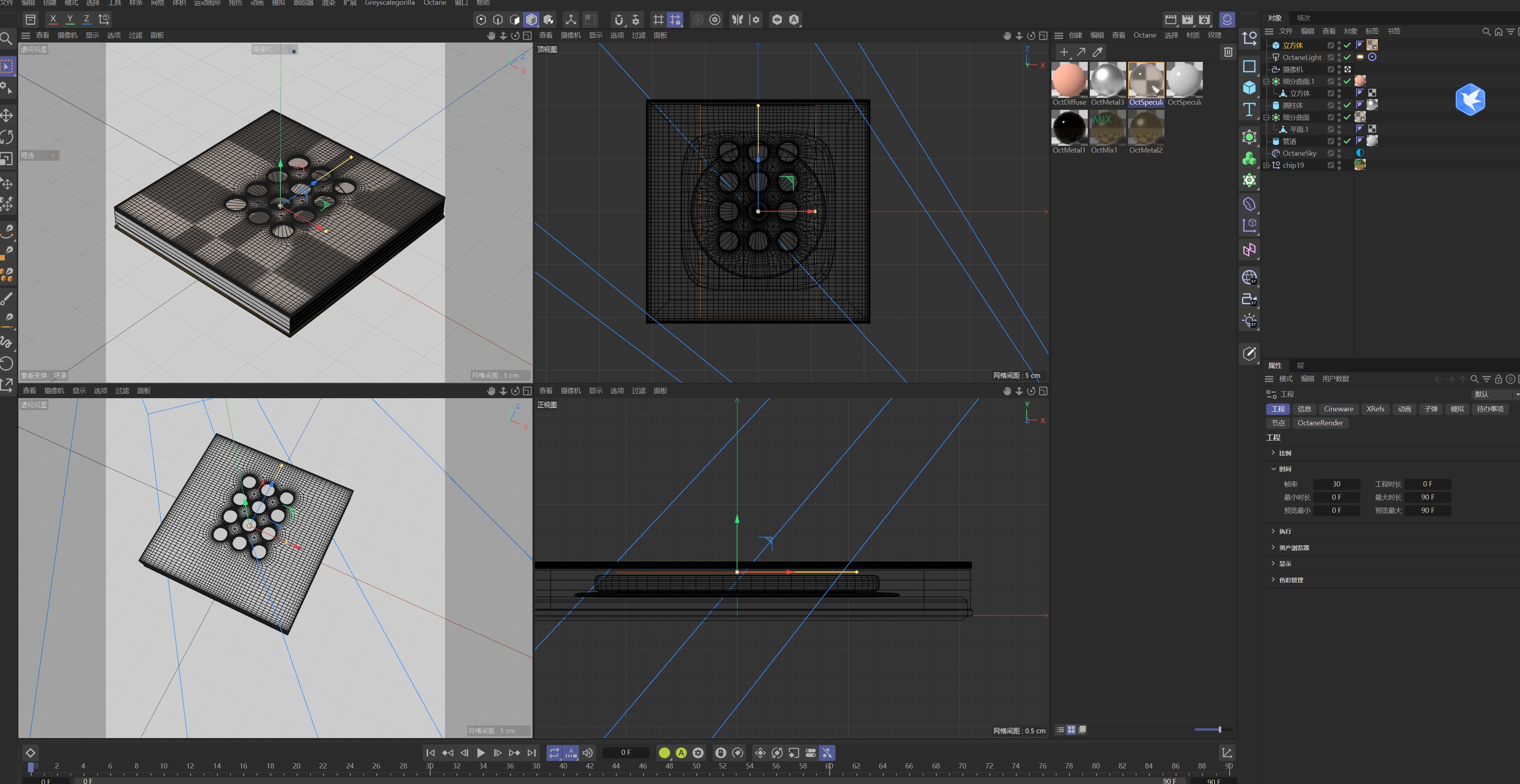

Supplement: Supplementary file 8 — Additional information on figures [file 41467_2024_48884_MOESM8_ESM.zip › Figure materials/optical images/Materials for Fig. 1g_upper right corner3.jpg]

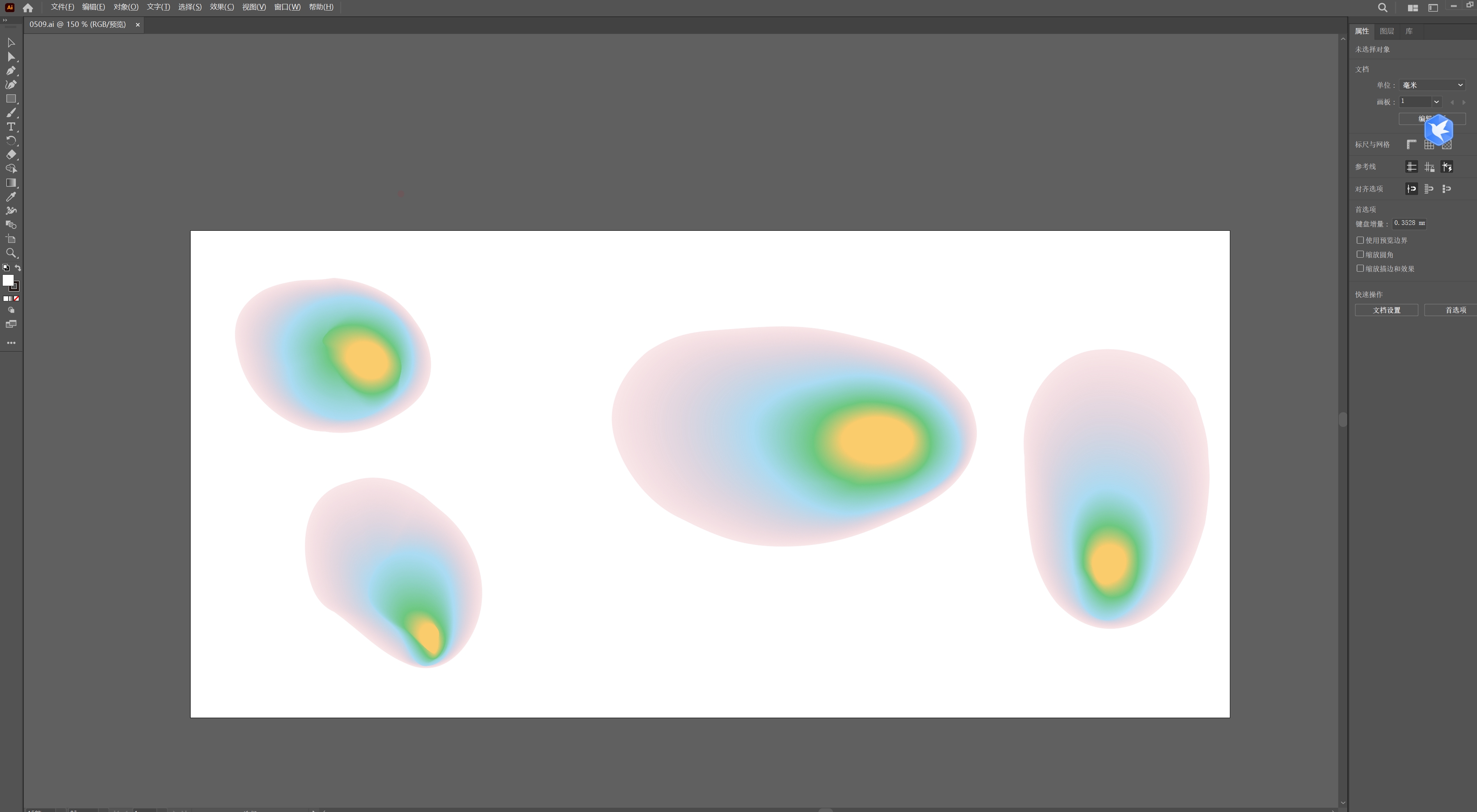

Supplement: Supplementary file 8 — Additional information on figures [file 41467_2024_48884_MOESM8_ESM.zip › Figure materials/optical images/materials for Fig. 5a.jpg]

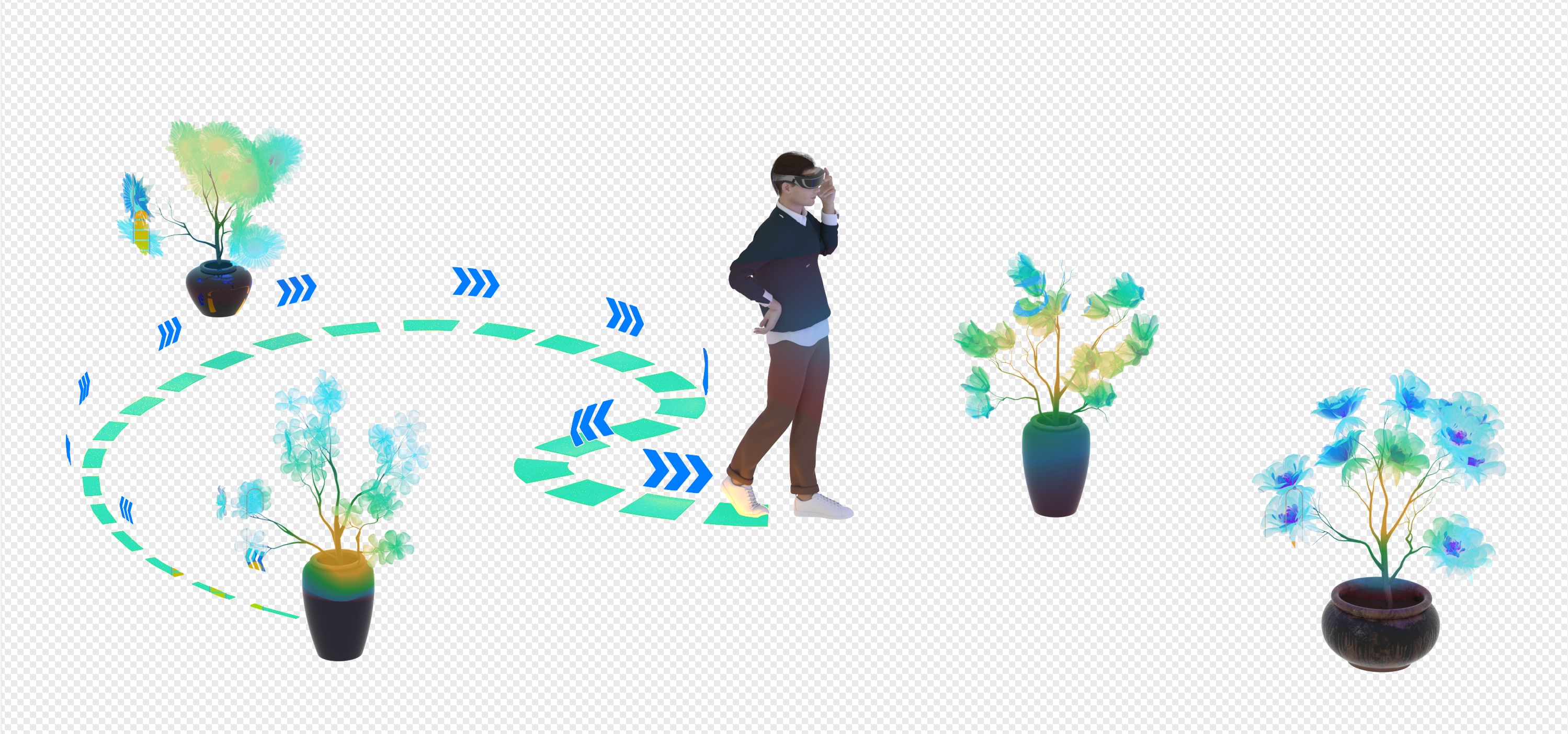

Supplement: Supplementary file 8 — Additional information on figures [file 41467_2024_48884_MOESM8_ESM.zip › Figure materials/optical images/materials for Fig. 5a_combination.jpg]

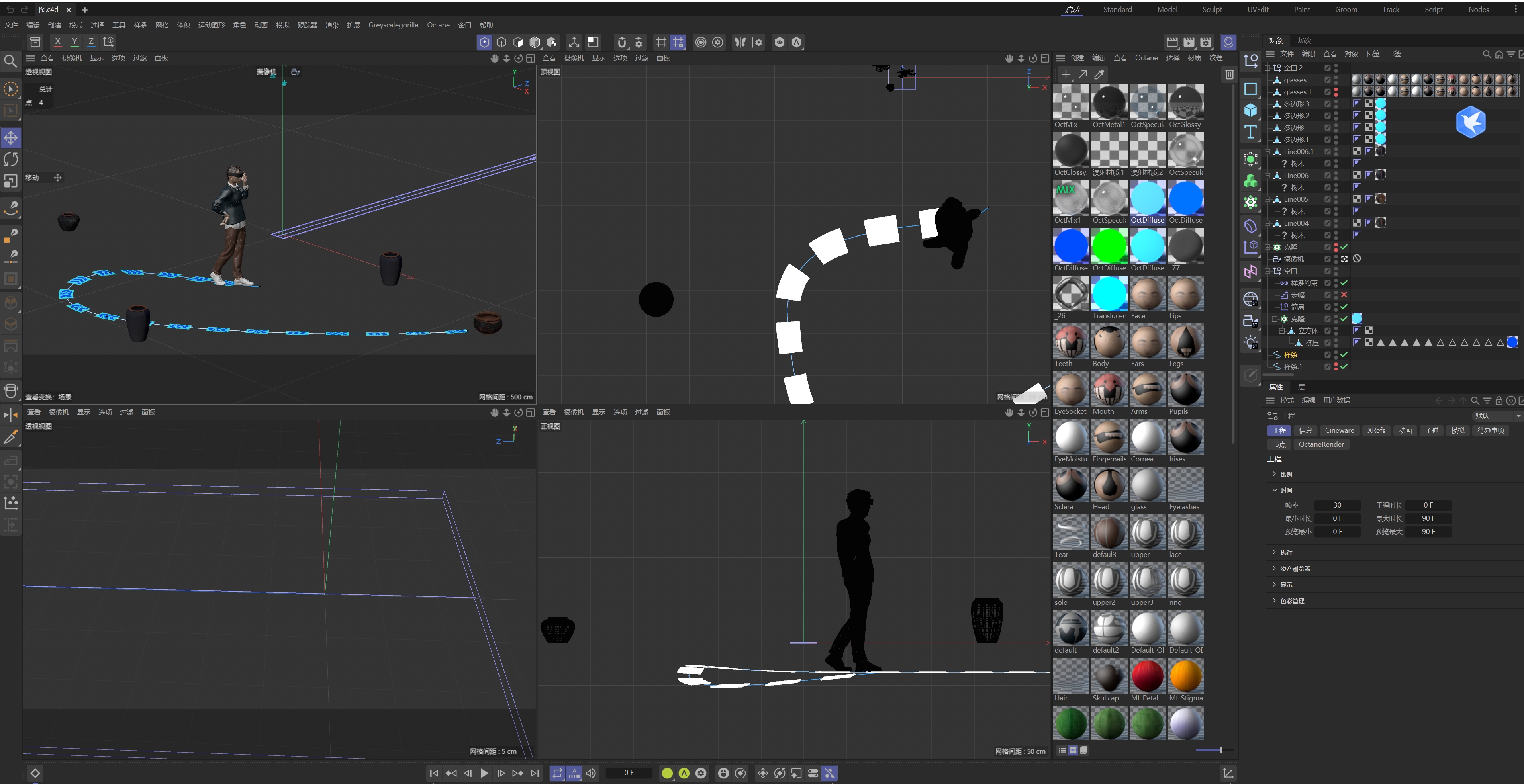

Supplement: Supplementary file 8 — Additional information on figures [file 41467_2024_48884_MOESM8_ESM.zip › Figure materials/optical images/materials for Fig. 5a_human routine.jpg]

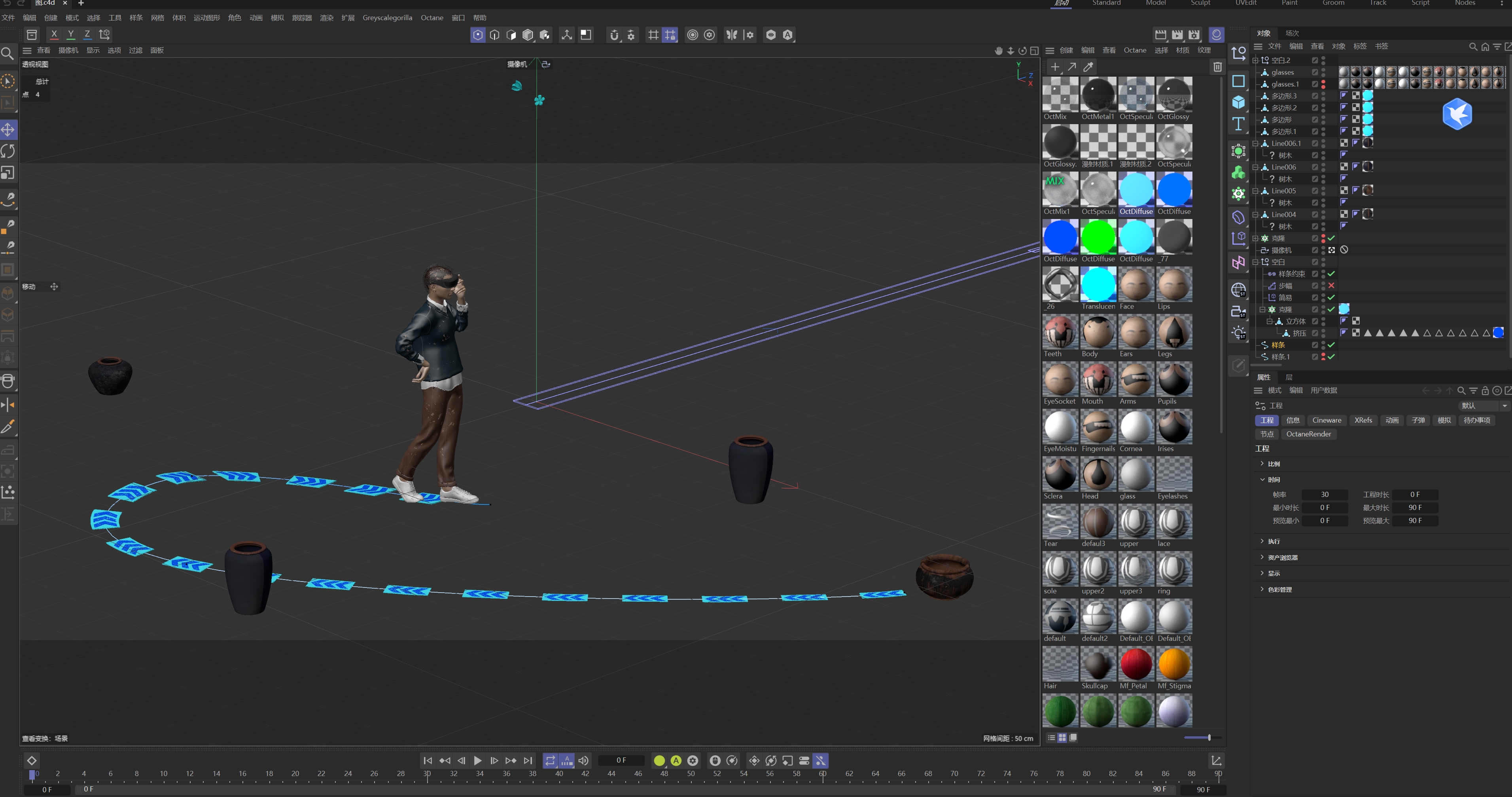

Supplement: Supplementary file 8 — Additional information on figures [file 41467_2024_48884_MOESM8_ESM.zip › Figure materials/optical images/materials for Fig. 5a_Human subject.jpg]
